# A Baseband Wireless Spectrum Hypervisor for Multiplexing Concurrent OFDM Signals

**DOI:** 10.3390/s20041101

**Published:** 2020-02-17

**Authors:** Felipe A. P. de Figueiredo, Ruben Mennes, Irfan Jabandžić, Xianjun Jiao, Ingrid Moerman

**Affiliations:** 1 IDLab, Department of Information Technology at Ghent University–IMEC, 9052 Ghent, Belgium; irfan.jabandzic@ugent.be (I.J.); xianjun.jiao@ugent.be (X.J.); ingrid.moerman@ugent.be (I.M.); 2 Instituto Nacional de Telecomunicações–INATEL, Santa Rita do Sapucaí 37540-000, MG, Brazil; 3 Department of Computer Science, University of Antwerp–IMEC, 2000 Antwerp, Belgium; ruben.mennes@uantwerpen.be

**Keywords:** radio virtualization, network densification, infrastructure sharing, multi-tenancy, cognitive radios, NB-IoT, MTC

## Abstract

The next generation of wireless and mobile networks will have to handle a significant increase in traffic load compared to the current ones. This situation calls for novel ways to increase the spectral efficiency. Therefore, in this paper, we propose a wireless spectrum hypervisor architecture that abstracts a radio frequency (RF) front-end into a configurable number of virtual RF front ends. The proposed architecture has the ability to enable flexible spectrum access in existing wireless and mobile networks, which is a challenging task due to the limited spectrum programmability, i.e., the capability a system has to change the spectral properties of a given signal to fit an arbitrary frequency allocation. The proposed architecture is a non-intrusive and highly optimized wireless hypervisor that multiplexes the signals of several different and concurrent multi-carrier-based radio access technologies with numerologies that are multiple integers of one another, which are also referred in our work as radio access technologies with correlated numerology. For example, the proposed architecture can multiplex the signals of several Wi-Fi access points, several LTE base stations, several WiMAX base stations, etc. As it able to multiplex the signals of radio access technologies with correlated numerology, it can, for instance, multiplex the signals of LTE, 5G-NR and NB-IoT base stations. It abstracts a radio frequency front-end into a configurable number of virtual RF front ends, making it possible for such different technologies to share the same RF front-end and consequently reduce the costs and increasing the spectral efficiency by employing densification, once several networks share the same infrastructure or by dynamically accessing free chunks of spectrum. Therefore, the main goal of the proposed approach is to improve spectral efficiency by efficiently using vacant gaps in congested spectrum bandwidths or adopting network densification through infrastructure sharing. We demonstrate mathematically how our proposed approach works and present several simulation results proving its functionality and efficiency. Additionally, we designed and implemented an open-source and free proof of concept prototype of the proposed architecture, which can be used by researchers and developers to run experiments or extend the concept to other applications. We present several experimental results used to validate the proposed prototype. We demonstrate that the prototype can easily handle up to 12 concurrent physical layers.

## 1. Introduction

According to [1], the average mobile connection speed in 2016 was 6.8 Mbps and it is forecast to grow at a compound annual growth rate (CAGR) of 24.4%, reaching nearly 20.4 Mbps by 2021. By 2020 it is forecast that there will be 11.6 billion mobile-connected devices, including Machine to Machine (M2M) modules [1]. Reports like [2] state that future mobile networks will need to support 1 million connections per square kilometer and up to a total of 100 billion connections in total.

The research and development of future mobile networks (i.e., next generation mobile networks) has been derived by several novel breakthroughs in a myriad of areas. The recent advancements of technologies such as licensed assisted access, carrier aggregation, massive multiple-input-multiple-output (massive MIMO), cooperative spectrum sensing, beamforming techniques, compressive sensing, machine learning, etc., have provided exciting and encouraging approaches to address the forecast increase in number of connections and their respective speeds [3,4,5,6,7]. These demanding requirements pose daunting challenges to the design of both base stations and user equipment. In particular, at the user equipment side, these new requirements bring a series of daring implementation issues due to limited design budget and hardware resources imposed to the implementation of those devices [6,8].

To support the expected growth in capacity, the forecast for the future mobile networks, the area throughput, i.e., bit/s/km${}^{2}$, needs to be increased. Some ways to improve area throughput in cellular networks include densification of existing cellular networks using extra-small cells, provision of peer-to-peer (P2P) communications, multi-tier heterogeneous networks, full-duplex communication, massive MIMO, millimeter-wave technologies, cognitive radios, beam division multiple access (BDMA), cloud-based radio access networks (CRAN) and wireless networks virtualization (WNV) [3,4,5,6].

One of the key features to achieve the forecast high data rates is the dense deployment of remote radio heads (RRHs). RRH is the name given to the RF front-end in mobile networks and encompasses the BS’s RF circuitry plus analog-to-digital/digital-to-analog converters and up/down converters. The dense deployment of RRHs can be achieved, with a relatively lower cost when compared to the deployment of several physical RRHs, by multiplexing several different signals at a single RRH and transmitting them over several virtual RRHs. Carrier aggregation is one of the usage examples, where a base station (BS) increases its capacity by allocating more spectrum bandwidth. Infrastructure sharing is another usage example, where densification is achieved by sharing already deployed pieces of equipment, such as the RRH, among several wireless or mobile networks [8,9].

Infrastructure sharing has the advantage of reducing capital expenditure. It is well known that the deployment of cellular or wireless networks is expensive, and raising the capital for that effort is quite difficult as the operators always want to make the most out of the already deployed infrastructure. Therefore, in order to obtain a better return on the costs related to installation and maintenance of mobile or wireless infrastructure, it would be less expensive to share the already (or newly) deployed infrastructure with other operators than to build overlapping and concurrent infrastructure [10].

This way, RF front-end virtualization becomes very useful for flexible spectrum access, once it allows several physical layers (PHYs) to concurrently share a single physical wide-band RF front-end. Some direct consequences of RF front-end virtualization are the reduction in the required physical space for deployment (only a single wide-band RRH is required), energy consumption and price (reduction in redundancy) for the infrastructure provider.

**Figure 1:**
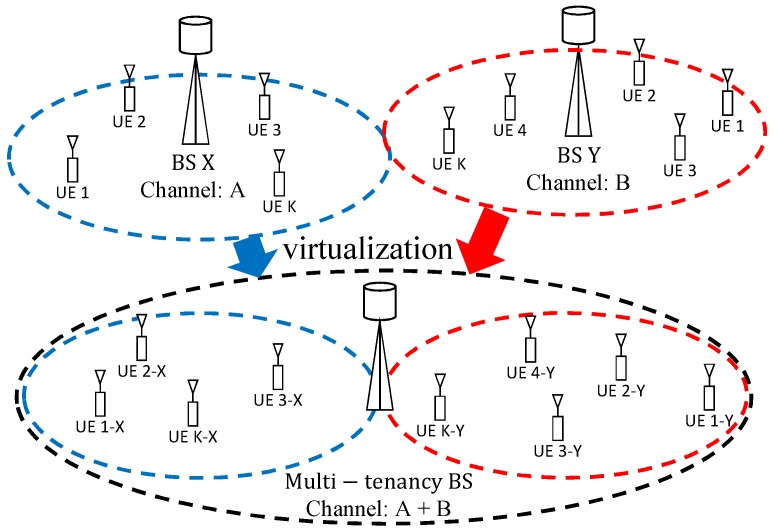
One exemplary use case for RF front-end virtualization: A single BS that emulates multiple virtual BSs. Here, subscribers from different networks associate with their corresponding virtual BS by using the same underlying infrastructure.

Figure 1 shows an example of a use case where RF front-end virtualization is used in a multi-tenancy application. In this example, two BSs, which are deployed at the same location, use different channels and have totally independent infrastructure (i.e., equipment), are replaced by a single multi-tenancy BS that emulates, through RF front-end virtualization, two BSs where subscribers from the two different networks associate with their corresponding virtual BS.

Another way of achieving such demanding rates is through better use of the available and already underused spectrum bands. In general, due to the presence of primary and secondary users in shared licensed bands and of competing (i.e., opportunistic) users in unlicensed bands, the available spectrum for users in a cognitive radio environment is fragmented and its use is intermittent, i.e., the available spectrum is split into non-contiguous chunks and is not used all the time. This intermittent and fragmented spectrum availability calls for a flexible and agile transmission scheme of the desired signals [11]. The concurrent transmission of several narrow orthogonal frequency-division multiplexing (OFDM)-based signals allows for the selective use of the available chunks of spectrum, which enhances the spectrum use, consequently improving the area throughput.

Therefore, in this work, we propose a non-intrusive and highly optimized wireless hypervisor architecture for software-defined radios (SDRs) that ensures coexistence, isolation, and programmability for multi-carrier-based systems (i.e., OFDM) with numerologies that are multiple integers of one another, i.e., systems that have subcarrier spacings that are integer multiples of the smallest adopted frequency spacing and cyclic prefix (CP) lengths that are integer multiples of the maximum possible number of multiplexed signals. The proposed architecture is meant for multiplexing concurrent signals of the same RAT or correlated RATs (e.g., LTE/LTE, LTE/NB-IoT, LTE/5G-NR, 5G-NR/5G-NR, NB-IOT/NB-IoT, Wi-Fi/Wi-Fi, ZigBee/ZigBee, etc.). We focus on supporting OFDM-based systems once such waveform is still one of the most used and important ones being used even in 5G standards like the 5G New-Radio (NR) ones [12,13]. Therefore, we understand that OFDM will still be widely adopted and employed for a long time.

The proposed hypervisor is a generic architecture that can be used to serve not only Wi-Fi, LTE and NB-IoT-based sensors, i.e., Machine-type communication (MTC) devices, but also serve other kinds of Human-type communication (HTC) devices. The main idea behind the hypervisor is the ability of having several Wi-Fi routers, LTE/NB-IoT or any other OFDM-based radio access technology (RAT) base stations sharing the same RF front-end.

We show that with the proposed architecture, spectrum programmability (i.e., the ability to program/change frequency, bandwidth and gain settings) can be decoupled from physical layer (PHY) processing and delegated to a virtualization layer, (i.e., the wireless hypervisor) which is added between a set of virtual PHYs (vPHYs) and the hardware/physical radio frequency (RF) front-end. The proposed solution can be applied to both approaches mentioned above of achieving higher area throughput.

We demonstrate that spectrum allocation can be decoupled from the PHY (or baseband processing) layer. In this way, the proposed hypervisor architecture supports flexible spectrum bandwidth allocation by creating a new layer located between several vPHYs and the physical RF front-end. The hypervisor dynamically maps the modulated signals of several vPHYs into configurable chunks of spectrum, before sending the resulting multiplexed signal of the vPHYs to the RF front-end. The hypervisor layer, which can be seen as a spectrum mapping/allocation layer, abstracts the underlying spectrum bandwidth dynamics and provides the vPHYs with a contiguous or non-contiguous (depending on the application of the proposed architecture) set of frequency subcarriers (i.e., virtual spectrum bandwidth), where the desired spectrum bandwidth can be predefined (by operators for instance) or requested in an online basis by the vPHY itself.

Additionally, we present an open-source and free proof of concept prototype that was developed to assess the performance of proposed spectrum hypervisor architecture in real-world experiments. The source code is available at [14] and can be used by researchers and developers to easily run experiments or extend the concept to other applications.

The remainder of the work is organized as follows. In Section 2, we present some aspects of hardware virtualization. In Section 3, we describe, mathematically, how we multiplex several OFDM signals. Next, in Section 4, we list and discuss some important use cases where the proposed architecture can be employed. In Section 5 we present and compare some related pieces of work on virtualization. Then, in Section 6, we describe the proof of concept prototype developed to assess the feasibility of the proposed architecture. In Section 7, some simulation results are presented showing the performance of the proposed architecture. Next, in Section 8, we present several experimental results obtained with the prototype. Finally, in Section 9, we conclude our work with some conclusions and future work.

## 2. Hardware Virtualization

Hardware (in our case, the RF front-end) virtualization is achieved by means of hypervisors [15,16]. A hypervisor is a hardware virtualization technique that abstracts, i.e., isolates, multiple concurrent software radio protocol stacks, also known as software-defined radios (SDR), of RATs with correlated numerology from the underlying radio hardware i.e., RF front-end. It allows multiple radio stacks to run on top of a single piece of radio hardware at the same time, i.e., concurrently, such that each protocol stack appears to have its own RF front-end (virtual RF front-end) that can be operated independently. One advantage is that wireless hypervisors help to maximize the effective use of the deployed infrastructure (i.e., servers, RF front ends, etc.).

**Figure 2:**
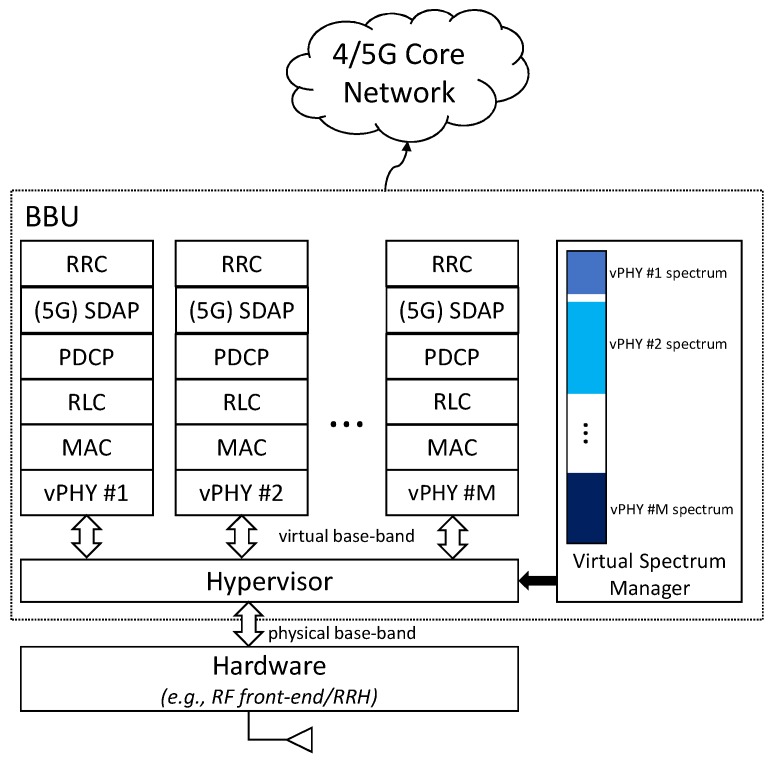
High-level view of a hardware hypervisor in 4G or 5G-like networks.

The hypervisor is the mechanism allowing the seamless sharing of a particular resource by meeting three key requirements: abstraction, programmability, and isolation. The proposed solution addresses each of these requirements as shown in Figure 2 and briefly described next.

- **Abstraction**: this feature hides the underlying hardware characteristics and establishes simplified interfaces for accessing and sharing the hardware resources. This feature allows the clients of the hypervisor architecture to use it with no change to their upper layer radio stack, i.e., from the MAC layer upwards. As shown in Figure 2, the proposed architecture provides concurrent access to multiple virtual RF front ends, which are exposed to clients through several vPHYs. The proposed architecture can be supported by inexpensive RF front ends (e.g., commodity SDR equipment) or expensive wide-band RRHs. The wireless hypervisor ensures that multiple vPHYs can concurrently coexist on top of the same physical RF front-end. The vPHYs are the point of access of the radio stacks to the virtual RF front ends. Along with the hypervisor, the vPHYs provide a computationally efficient way of multiplexing several baseband signals into a wide-band signal, which consequently splits the spectrum bandwidth provided by the physical front-end. The proposed architecture supports the operation of multiple concurrent vPHYs, implementing totally different air-interfaces (as long as they are OFDM-based signals and have subcarrier spacings that are integer multiples of the smallest adopted spacing) with diverse processing constraints, channel bandwidths, medium access schemes, etc.
- **Programmability**: the proposed wireless hypervisor has to provide the multiple radio stacks running on top of it the same level of programmability (or configurability) provided by the single physical RF front-end. This feature is addressed by allowing the multiple radio stacks to configure different center frequencies, bandwidths and transmission/reception gains for individual virtual RF front ends, i.e., the virtual RF front ends must provide the same set of functionalities provided by the physical RF front-end.
- **Isolation**: the proposed wireless hypervisor must make sure that any configuration or wrong configuration does not affect or cause interference to any other collocated radio stack. Isolation is the fundamental requirement that guarantees fault tolerance, security, and privacy to the multiple radio stacks running (i.e., coexisting) on top of the same physical RF front-end [16]. This feature is enforced across multiple clients by providing them predefined access-points and bandwidths that do not overlap.

Figure 2 shows one out of several possible multi-tenancy uses for the proposed architecture, where it is employed in 4G or 5G-like mobile networks. Our proposed approach is generic enough to be applied to both 4G or 5G standards or even to a mix of the two standards, since they both use OFDM-based PHYs. However, it could also be, for example, employed by Internet Service Providers (ISP) aiming at sharing their already deployed Wi-Fi access-points (or hot-spots) at locations such as airports, cafes and common shopping areas.

**Figure 3:**
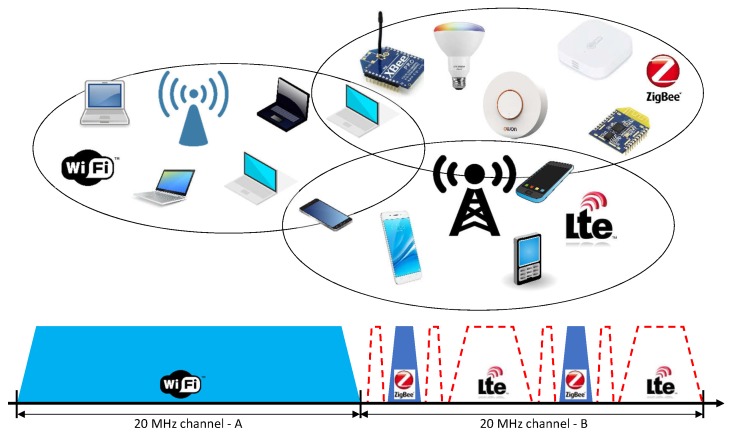
Flexible and dynamic spectrum access example. Communications spectrum bandwidths can be flexibly and dynamically changed according to the availability of spectrum as well as application requirements.

Figure 3 depicts yet another use case example regarding dynamic and flexible spectrum access. In the current wireless communications networks, channels have fixed central frequencies and bandwidths. Such static channel allocation causes spectrum fragmentation, lowering the spectrum use efficiency. Figure 3 shows a common coexistence scenario in industrial, scientific and medical (ISM) bands. In these bands it is common to have the coexistence of several different technologies, such as Wi-Fi, IEEE 802.15.4 (e.g., ZigBee), LTE-U, etc. [17]. In the figure, two IEEE 802.15.4 networks occupy two channels that overlap with a 20 MHz-wide Wi-Fi channel. As can be seen, the two IEEE 802.15.4 networks may render the channel unusable for Wi-Fi communications due to the narrow-band interference caused by the two IEEE 802.15.4 networks. However, the remaining fragmented spectrum in that channel could be used by a flexible and dynamic system, as depicted by the red-dashed lines in Figure 3. As it is shown, the spectrum gaps created by the IEEE 802.15.4 transmissions could be used to establish LTE-like networks (e.g., LTE-U, 5G-NR, NB-IoT, etc.) or any other kind of OFDM-based network. This flexible and dynamic spectrum usage completely removes the concept of predefined and static channel allocations. Based on this approach, frequencies and bandwidths can be dynamically allocated based on the availability of spectrum and the requirements of the applications.

## 3. Multiplexing Concurrent OFDM Signals

**Figure 4:**
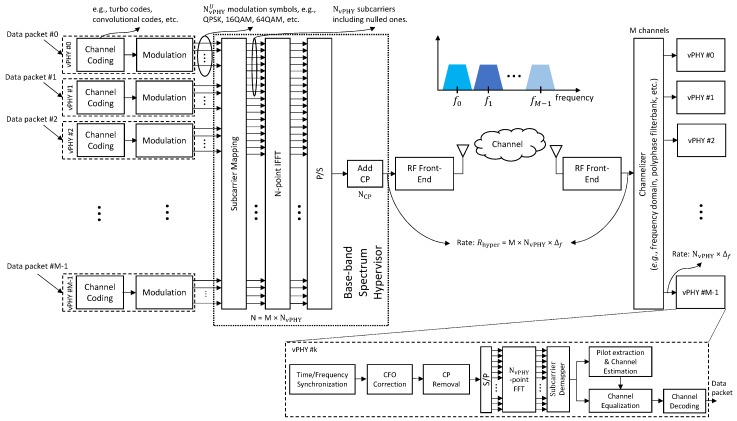
Architecture of the proposed baseband wireless hypervisor.

In this section, we present a mathematical analysis of the working of the hypervisor proposed in this work and depicted in Figure 4.

For this analysis, we consider that complex modulated samples, $s_{i}$, carrying information are grouped into several narrow sub-bands, $B_{k}\left(i\right)$, which are composed of $N_{\mathrm{vPHY}}$ subcarriers. The complex samples, $s_{i}$, are symbols drawn from a digital modulation scheme such as BPSK, QPSK, 16QAM, etc. A maximum number of $M=⎣N/N_{\mathrm{vPHY}}⎦$ narrow sub-bands, containing $N_{\mathrm{vPHY}}$ subcarriers can be used, where *N* is the length of the IFFT used in the hypervisor and ⎣c⎦ is known as the floor operator and gives the largest integer value that is less than or equal to *c*. Out of the $N_{\mathrm{vPHY}}$ subcarriers, a narrow sub-band might have, $N_{\mathrm{vPHY}}^{U}$ carrying useful information, i.e., the modulation symbols $s_{i}$, one Direct Current (DC) subcarrier, $N_{\mathrm{vPHY}}^{DC}$, which is set to zero, and $N_{\mathrm{vPHY}}^{Null}$ subcarriers that are also set to zero. The nulled subcarriers can be used as frequency guard bands at both edges of the narrow sub-band, allowing for the straightforward realization of anti-aliasing filters. On the other hand, the DC subcarrier allows the use of the simpler and cheaper direct-conversion, also known as zero intermediate-frequency (IF), RF front ends. The narrow sub-band signal can be defined as

(1) $$B_{k}\left(i\right)=\left\{\begin{array}{cc} 0, & i=0,\\ s_{i}, & 1\leq i\leq \frac{N_{\mathrm{vPHY}}^{U}}{2},\\ 0, & \left(\frac{N_{\mathrm{vPHY}}^{U}}{2}\right)+1\leq i\leq \left(\frac{N_{\mathrm{vPHY}}^{U}}{2}\right)+N_{\mathrm{vPHY}}^{Null},\\ s_{i}, & \left(\frac{N_{\mathrm{vPHY}}^{U}}{2}\right)+N_{\mathrm{vPHY}}^{Null}+1\leq i\leq N_{\mathrm{vPHY}}-1. \end{array}\right.$$

For the sake of clarity, we consider that *N* is split into *M* sub-bands with $N_{\mathrm{vPHY}}$ subcarriers and that the subcarrier mapping block in the hypervisor maps complex samples into contiguous sub-bands. Please note that the mapping of the *M* sub-bands, $B_{k}\left(i\right)$, into the IFFT subcarriers/bins corresponds to a frequency translation (mixing) of the sub-band signal, which is centered around 0 [Hz], to a frequency offset defined by the bin number times the subcarrier spacing, $\Delta_{f}=R_{\mathrm{hyper}}/N$, where $R_{\mathrm{hyper}}$ is the sampling rate used by the hypervisor. The hypervisor’s sampling rate, $R_{\mathrm{hyper}}$, defines the amount of physical spectrum bandwidth that it can virtualize.

The set, S(i), containing the concatenation of all the *M* narrow sub-bands, $B_{k}\left(i\right)$, and of length *N* is transformed into time domain using an IFFT of size *N* as

(2) $$\begin{array}{cc} s(n) & =\frac{1}{N}\sum_{i=0}^{N-1}S\left(i\right)e^{-j2\pi in/N}\\ \; & =\frac{1}{N}\sum_{k=0}^{M-1}\sum_{i^{\prime }=0}^{N_{\mathrm{vPHY}}-1}B_{k}\left(i^{\prime }\right)e^{-j2\pi in/N}, \end{array}$$

where $i=i^{\prime }+kN_{\mathrm{vPHY}}$. Then, based on (2), the transmitted wide-band signal, $y_{\mathrm{wBB}}\left(n\right)$, including a gain factor and the appending of a cyclic prefix can be represented by

(3) $$\begin{array}{c} y_{\mathrm{wBB}}\left(n\right)=\frac{1}{N}\sum_{k=0}^{M-1}\sum_{i^{\prime }=0}^{N_{\mathrm{vPHY}}-1}\rho_{k}B_{k}\left(i^{\prime }\right)e^{-j2\pi (i^{\prime }+kN_{\mathrm{vPHY}})n/N},\\ -N_{CP}\leq n\leq N-1, \end{array}$$

where $\rho_{k}$ is the frequency amplification factor (or frequency gain), varying from 0 to 1, applied individually to each sub-band and $N_{CP}$ is the CP length, which has to be an integer multiple of *M*. As can be noticed, $y_{\mathrm{wBB}}\left(n\right)$ is a wide-band signal containing all the *M* multiplexed vPHY signals. As the final step in the transmission chain, the wide-band signal $y_{\mathrm{wBB}}\left(n\right)$ is sent to the RF front-end, which will translate the signal into the desired pass-band frequency.

**Figure 5:**
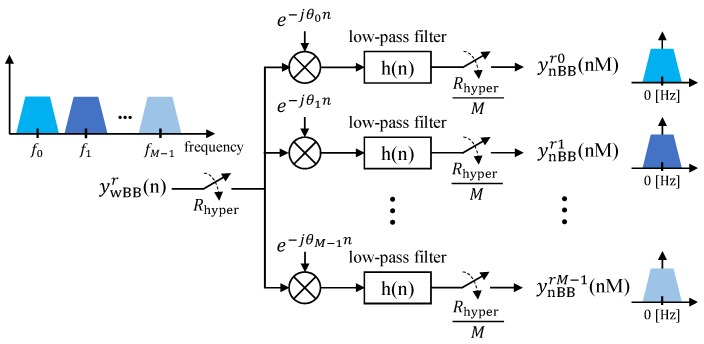
Architecture of a conventional channelizer: frequency offset to baseband, low-pass filters, and down-samplers.

At the receiver side, the wide pass-band signal is translated into a wide baseband signal by the receiver RF front-end. Here we do not consider any channel influence, i.e., the transmitted signal does not suffer from any impairment, and consequently, it is perfectly received at the receiver side. Next, after the frequency translation, the received wide baseband signal $y_{\mathrm{wBB}}^{r}\left(n\right)=y_{\mathrm{wBB}}\left(n\right)$, which contains the *M* multiplexed sub-band (narrow-band) signals, is digitally translated/split into *M* narrow baseband signals (each one centered around 0 [Hz]), bandwidth restricted by low-pass digital Finite Impulse Response (FIR) filters, and finally, submitted to a sample rate reduction. These steps are depicted in Figure 5, which shows the architecture of a conventional channelizer as described in [18]. As shown in the figure, the output of the *k*-th channel, which is denoted by $y_{\mathrm{wBB}}^{r_{k}}$, prior to the down-sampling, is a convolution as defined by

(4) $$\begin{array}{cc} y_{\mathrm{wBB}}^{r_{k}}\left(n\right) & =\left[y_{\mathrm{wBB}}^{r}\left(n\right)e^{j\theta_{k}n}\right]⊛h\left(n\right)\\ \; & =\sum_{l=0}^{N_{\mathrm{FIR}}-1}y_{\mathrm{wBB}}^{r}\left(n-l\right)e^{j\theta_{k}\left(n-l\right)}h\left(l\right),\\ & 0\leq n<N+N_{\mathrm{FIR}}-1 \end{array}$$

where $N_{\mathrm{FIR}}$ is the length of the FIR filter, $\theta_{k}=\frac{2\pi \delta_{k}n}{N}$ is the angle corresponding to the digital frequency offset, and $\delta_{k}$ is the center frequency of the *k*-th narrow sub-band, $B_{k}$, in number of subcarrier spacings, $\Delta_{f}$. After substituting (3) into (4) we have

(5) $$\begin{array}{c} y_{\mathrm{wBB}}^{r_{k}}\left(n\right)=\sum_{l=0}^{N_{\mathrm{FIR}}-1}\frac{1}{N}\sum_{k=0}^{M-1}\sum_{i^{\prime }=0}^{N_{\mathrm{vPHY}}-1}\rho_{k}B_{k}\left(i^{\prime }\right)e^{\frac{-j2\pi \Theta_{i^{\prime },k}\left(n-l\right)}{N}}h\left(l\right),\\ -N_{CP}\leq n\leq N-1, \end{array}$$

where $\Theta_{i^{\prime },k}=\left(i^{\prime }+kN_{\mathrm{vPHY}}-\delta_{k}\right)\;\mathrm{mod}\;N$. The multiplication of $y_{\mathrm{wBB}}^{r}\left(n\right)$ by $e^{j\theta_{k}n}$ centers the *k*-th narrow sub-band, $B_{k}$, around 0 [Hz] and the convolution with the low-pass filter makes sure that only the signal components belonging to the *k*-th narrow sub-band go through it while the other sub-bands are filtered out. This way, (5) can be re-written as

(6) $$\begin{array}{c} y_{\mathrm{wBB}}^{r_{k}}\left(n\right)=\frac{1}{N}\sum_{i=0}^{N-1}\left\{\begin{array}{cc} \rho_{k}B_{k}\left(i\right)e^{\frac{-j2\pi ni}{N}}, & 0\leq i\leq \frac{N_{\mathrm{vPHY}}}{2},\\ \rho_{k}B_{k}\left(i\right)e^{\frac{-j2\pi ni}{N}}, & N-\frac{N_{\mathrm{vPHY}}}{2}+1\leq i\leq N-1,\\ 0, & \mathrm{otherwise}, \end{array}\right.\\ -N_{CP}\leq n\leq N-1. \end{array}$$

As can be seen by inspecting (6), out of *N* subcarriers/bins, only the lower and upper $\frac{N_{\mathrm{vPHY}}}{2}$ bins contain useful values, i.e., values different from zero. We consider in (6) that the filters have a perfect window-shaped frequency response, which is not true in practice but can be approximated by well-designed high order low-pass filters [19].

Next, the wide baseband representation of the *k*-th narrow sub-band signal, $y_{\mathrm{wBB}}^{r_{k}}\left(n\right)$, is subject to a down-sampling operation, as depicted in Figure 5, in order to have a narrow baseband representation of the sub-band signal, which can then be fed into a PHY for data decoding. After down-sampling, the signal expressed by (6), can be written as

(7) $$\begin{array}{c} y_{\mathrm{wBB}}^{r_{k}}\left(nM\right)=\frac{1}{N}\sum_{i=0}^{N-1}F\left(i\right)e^{\frac{-j2\pi ni}{N/M}},\;\;-N_{CP}\leq nM\leq N-1. \end{array}$$

where the function F(i) is defined as

(8) $$\begin{array}{c} F\left(i\right)=\left\{\begin{array}{cc} \rho_{k}B_{k}\left(i\right), & 0\leq i\leq \frac{N_{\mathrm{vPHY}}}{2},\\ \rho_{k}B_{k}\left(i\right), & N-\frac{N_{\mathrm{vPHY}}}{2}+1\leq i\leq N-1,\\ 0, & \mathrm{otherwise}. \end{array}\right. \end{array}$$

Next, after the down-sampling operation the complex periodic signal $e^{\frac{-j2\pi ni}{N}}$ has its periodicity reduced from *N* to N/M samples, $e^{\frac{-j2\pi ni}{N/M}}$, and therefore, we can rewrite (7) as

(9) $$\begin{array}{c} y_{\mathrm{wBB}}^{r_{k}}\left(nM\right)=\frac{1}{N}\left[\sum_{i=0}^{N_{\mathrm{vPHY}}-1}F\left(i\right)e^{\frac{-j2\pi ni}{N/M}}\right.\\ \left.+\sum_{i=N_{\mathrm{vPHY}}}^{2N_{\mathrm{vPHY}}-1}F\left(i\right)e^{\frac{-j2\pi ni}{N/M}}+\cdots +\sum_{i=\left(M-1\right)N_{\mathrm{vPHY}}}^{MN_{\mathrm{vPHY}}-1}F\left(i\right)e^{\frac{-j2\pi ni}{N/M}}\right]\\ =\frac{1}{N}\sum_{i=0}^{N_{\mathrm{vPHY}}-1}\left[\sum_{m=0}^{M-1}F(i+mN_{\mathrm{vPHY}})\right]e^{\frac{-j2\pi ni}{N/M}},\\ -N_{CP}\leq nM\leq N-1. \end{array}$$

By recalling the definition of F(i) given by (8), we realize that only half (i.e., $\frac{N_{\mathrm{vPHY}}}{2}$) of the first and last terms of the summation in (9) are different from 0, and therefore, it can be re-written as

(10) $$\begin{array}{c} y_{\mathrm{wBB}}^{r_{k}}\left(nM\right)=\frac{1}{N}\sum_{i=0}^{\frac{N_{\mathrm{vPHY}}}{2}}F\left(i\right)e^{\frac{-j2\pi ni}{N/M}}\\ +\frac{1}{N}\sum_{i=\frac{N_{\mathrm{vPHY}}}{2}+1}^{N_{\mathrm{vPHY}}-1}F\left(i+\left(M-1\right)N_{\mathrm{vPHY}}\right)e^{\frac{-j2\pi ni}{N/M}},\\ -N_{CP}\leq nM\leq N-1. \end{array}$$

Finally, by using (8) and remembering the fact that $N/M=N_{\mathrm{vPHY}}$, we show that the time-domain representation of the *k*-th narrow sub-band, $B_{k}\left(i\right)$, is recovered by the *k*-th branch of the channelizer (see Figure 5)

(11) $$\begin{array}{c} y_{\mathrm{nBB}}^{r_{k}}\left(n\right)=\frac{1}{N}\sum_{i=0}^{N_{\mathrm{vPHY}}-1}\rho_{k}B_{k}\left(i\right)e^{\frac{-j2\pi ni}{N_{\mathrm{vPHY}}}},\\ -N_{CP}^{\mathrm{vPHY}}\leq n\leq N_{\mathrm{vPHY}}-1, \end{array}$$

where $N_{CP}^{\mathrm{vPHY}}=N_{CP}/M$ is the cyclic prefix length of the vPHYs.

It is important to mention that this analysis is the same for any other narrow sub-band centered at any of the *N* subcarriers of the hypervisor’s IFFT module. Additionally, it is also important to notice that each one of the narrow sub-bands, which are multiplexed by the hypervisor, can have different widths. The only requirement is that the width of individual sub-bands is a divisible multiple of *N*. In this case, if a device is receiving more than one of the multiplexed narrow sub-band signals, then, it has to employ a non-uniform channelizer [20], as polyphase filter-bank channelizers only extract equally spaced spectrum chunks.

### Discussion

In this section, we discuss the proposed architecture, its functionalities, features, possible uses and limitation.

As mathematically shown earlier, the proposed wireless hypervisor provides a virtual and discretized (in steps of $\Delta_{f}=R_{\mathrm{hyper}}/N$) baseband spectrum abstraction layer to the several vPHY layers sitting on top of it. Its key function is the multiplexing, in the frequency domain, of several narrow baseband signals. It receives concurrent sets of modulated signals (e.g., BPSK, QPSK, M-QAM, etc.) from several vPHYs and maps these sets into continuous or non-contiguous subcarriers (i.e., spectrum) that will then be converted into time-domain by the *N*-point IFFT module and have a proper $N_{CP}$ samples long CP added to it. The digital signal processing carried out by the spectrum hypervisor transforms the sets of modulated vPHY signals into a wide baseband waveform signal that is appropriate for transmission, while keeping the concurrent sets of vPHY signals unchanged and isolated from each other.

The hypervisor supports data flows from multiple concurrent vPHYs and provides each one of them a virtual RF front-end, which can have the following settings configured independently: frequency-domain gain ($\rho_{k}$), frequency location (given by the mapping of the sub-band into the hypervisor’s IFFT) and bandwidth of the vPHY ($N_{\mathrm{vPHY}}$). Regarding the gain settings for each vPHY, as there is only one physical RF amplifier, the gain of the amplifier is set to a reasonable level (a level that avoids saturating the signal) and the independent vPHYs can set what we called frequency gain ($\rho_{k}$), which is a gain applied to the frequency-domain signal and corresponds to a percentage (i.e., 0 up to 100 %) of the RF amplifier’s current gain.

Access to the wireless hypervisor is exposed through the vPHYs. The vPHYs work the same way as regular OFDM-based PHY layers (e.g., Wi-Fi, WiMAX, LTE, 5G-NR, NB-IoT, etc.) being the only exception the removal of the OFDM modulation part. In the proposed architecture (see Figure 4), OFDM modulation is now executed by the wireless hypervisor, which efficiently multiplexes the baseband signal of several vPHYs into a single wide baseband signal. By removing and transferring the OFDM modulation of all PHYs to the hypervisor, we optimize the whole baseband processing performance by avoiding redundant/unnecessary, for example FFT and IFFT, operations that would be required to multiplex several concurrent regular PHY signals into a wide baseband signal [21,22,23]. The down-side of our approach is that it can only operate with multi-carrier-based (i.e., OFDM) signals.

At the receiver side, different receiving approaches might be followed, depending on how the proposed hypervisor is employed to generate the wide baseband signal. The first approach refers to a communications connection between two devices (i.e., a point-to-point connection). In this approach, as a first step, the received wide baseband signal coming from the RF front-end is split by a channelizer into equally or unequally (i.e., non-uniform) spaced spectrum chunks and then, the down-converted and down-sampled narrow sub-band signals are fed into the narrow-band PHY receivers running on the device. The second approach is similar to the first one, where the only difference is that the signal coming from the RF receiver front-end is not split into narrow sub-bands. Here the wide-band received signal is fed directly into the OFDM demodulator of a corresponding wide-band vPHY. In the third approach, several independent, distributed and narrow-band devices have their RF receiver front ends tuned to the center frequency and transmission bandwidth of each one of the transmitted vPHY signals. The fourth approach represents a mix of the previous three approaches, where there could be wide-band devices tuning to more than one transmitted narrow-band vPHY signal while other narrow-band devices would only be tuning to individual transmitted narrow-band vPHY signals. In all the approaches mentioned above, the signal multiplexing carried out at the transmitter side is transparent to the PHY receivers, meaning that the radio stack at the receivers does not need to be modified.

As a limitation, the proposed hypervisor architecture supports the operation of multiple concurrent RATs as long as they are OFDM-based signals, have subcarrier spacings that are integer multiples of the smallest adopted frequency spacing and CP lengths that are integer multiples of the maximum number of vPHYs, which is given by *M*, i.e., RATs with numerologies that are multiple integers of one another, which are also referred here as RATs with correlated numerology. This limitation was chosen at design-time so that the proposed architecture could get rid of several digital signal processing tasks (e.g., up/down-conversion, filtering, etc.) necessary by a BS to handle uncorrelated RAT numerologies (e.g., LTE, Wi-Fi, ZigBee, etc.) but that would increase the processing time and complexity of a given solution for multiplexing these RATs. Therefore, we have chosen this limitation for the sake of processing time and complexity optimization. Our approach is novel when compared to other ones (please, refer to Section 5) that support the multiplexing of RATs with uncorrelated numerology by performing several additional digital signal processing tasks that affect the processing performance of a BS, especially when the number of multiplexing signals increases. With our proposed approach, we wanted to focus on correlated RATs (i.e., RATs with numerologies that are multiple integers of one another) and with that have an optimized architecture for such RATs. Therefore, based on the correlated numerology limitation, it is not possible to employ the proposed architecture to multiplex the signal of RAT technologies with uncorrelated numerologies such as LTE, Wi-Fi and ZigBee. As mentioned in the use cases given in Section 4, the proposed architecture is meant for multiplexing concurrent signals of the same RAT or correlated RATs (e.g., LTE/LTE, LTE/NB-IoT, NB-IOT/NB-IoT, Wi-Fi/Wi-Fi, ZigBee/ZigBee, etc.). Consequently, if the different RATs have correlated numerologies, then, as analytically proved in Section 3, these different and concurrent RAT signals can be orthogonally multiplexed successfully.

## 4. Use Cases

In this section, we present and discuss some possible use cases for the proposed architecture.

### 4.1. Dynamic Spectrum Access

The Federal Communications Commission (FCC) has reported that licensed spectrum bandwidths are greatly underused [24]. One of the examples mentioned in the report is the TV spectrum, which has one of the lowest use rates. That spectrum band is in most cases left totally unused in areas not so populated (e.g., rural areas), and due to the fact that it is referred to as TV whitespace. Based on this under-use of spectrum originally meant only for TV broadcasting, regulators are opening up this previously licensed spectrum for unlicensed use [25]. TV whitespaces for unlicensed use brought about a revolution to cognitive radio, spectrum sensing as well as to dynamic spectrum access [26]. TV whitespaces allow for the opportunistic use of vacant TV channels for data communications [27,28].

Therefore, based on the opportunistic use of vacant spectrum, one of the use cases of the proposed wireless hypervisor is instantiating it as a Non-Contiguous Orthogonal Frequency-Division Multiplexing (NC-OFDM) PHY, where one or several vPHYs could, based on spectrum availability/occupancy, have their data mapped into subcarriers that do not interfere/overlap with primary users in a Dynamic Spectrum Access (DSA) scheme, and thereby enabling efficient use of the spectrum [11]. For this use case, an NC-OFDM PHY node would employ the proposed hypervisor architecture as a wide-band OFDM modulator, where the subcarrier mapping module depicted in Figure 4 maps the modulated symbols only into subcarriers that correspond to vacant spectrum, i.e., not being used by the primary or other users. At the receiver side, the signal is demodulated and decoded as a wide-band NC-OFDM signal where some subcarriers had their values set to zero. The PHY receiver for this signal is depicted in the bottom right corner of Figure 4, and is nothing but a generic OFDM demodulator/decoder where the subcarrier demapper module needs to know beforehand which subcarriers are active during a given transmission interval [11].

**Figure 6:**
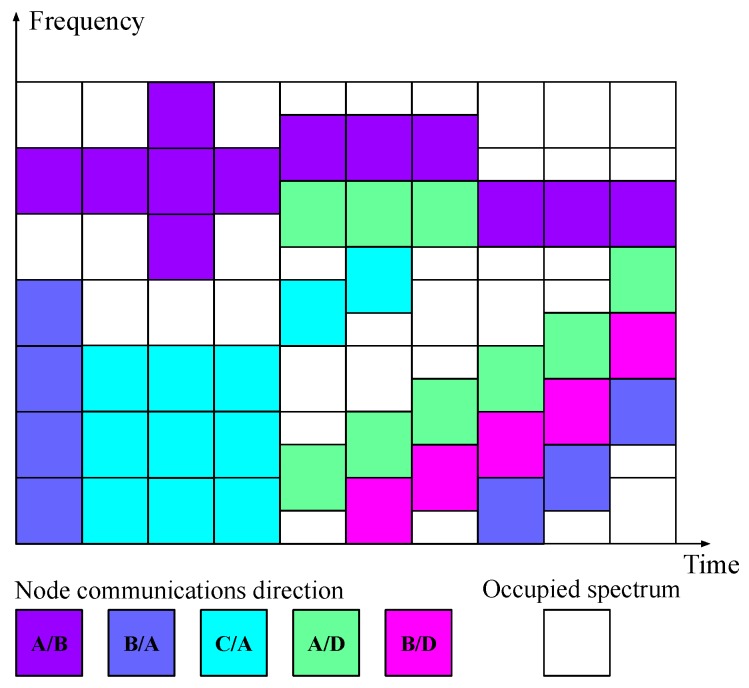
Snapshot of the spectrum usage in the TV whitespace.

Figure 6 depicts a possible snapshot of the spectrum usage in the TV whitespace when the proposed architecture is used as a dynamic packet-based PHY layer with allocated bandwidth being dynamically changed according to vacant spectrum. In this example, the PHY layer establishes concurrent/simultaneous communications with several nodes. As can be seen, the channel’s center frequency and allocated transmission bandwidth are changed throughout time, depending on spectrum availability and/or traffic load.

Another use case for the proposed architecture is in the implementation of a spectrum-sharing scheme between wireless operators and Fixed Satellite Services (FSS) in the Citizens Broadband Radio Service (CBRS) band. FSS stations must share spectrum with new entrant wireless operations, while the entrant networks must ensure that the interference that they introduce to the incumbent FSS remains below a specified threshold [29,30]. The aim with the launch of CBRS in the USA is that wireless systems should dynamically share the spectrum among them [30]. In this use case, the proposed architecture could easily adapt its transmission bandwidth to the available (either continuous or discontinuous) spectrum at any specific time. This use case is similar to the one described by Figure 6. Mainly aligned with this use case, DARPA, the Defense Advanced Research Projects Agency from the United States, has started the Spectrum Collaboration Challenge with the aim to encourage research and development of smarter/more intelligent coexistence and collaboration techniques of heterogeneous networks in the same wireless spectrum bands [31,32]. One of the examples they have been advocating for is the adoption of such spectrum-sharing technologies in the CBRS band [33,34].

### 4.2. Network Densification

The capacity of an additive white Gaussian noise (AWGN) channel is given by the following equation [9]

(12) $$R<C=m\left(\frac{W}{n}\right)\log_{2}\left(1+\frac{S}{I+N}\right),$$

where *W* is the BS allocated bandwidth, *n* denotes the BS load factor (i.e., the number of users sharing the given BS), *m* is the spatial multiplexing factor (i.e., it denotes the number of spatial data streams connecting the BS and devices), *S* gives the signal power and *I* and *N* represent the interference and power noise, respectively, experienced at the receiver side. After analyzing the equation, it is possible to see that the capacity can be increased by decreasing the BS load factor, which can be attained through cell splitting. Cell splitting involves deploying a larger number of BSs and making sure that the user traffic is evenly distributed among all the deployed BSs [9]. A possible consequence of cell splitting/densification is that it might improve the signal power, as the users could now be closer to the new deployed BS and consequently experiencing a reduced path-loss.

The dense deployment of infrastructure is a precondition for cutting the BS load factor *n* down in (12). However, the deployment of additional BSs entails significant costs and detailed site survey/planning [35]. Therefore, another use case, which represents a very important application for the proposed architecture, is instantiating the proposed architecture as an RF front-end multiplexer, where several vPHYs share a single physical RF front-end. This could be employed in multi-tenancy cases [21], where a cellular network infrastructure provider shares their owned infrastructure and/or spectrum with mobile virtual network operators (MVNOs) or vertical markets such as energy, automotive, city management, food and agriculture, health-care, government, public transportation, manufacturing, etc. In this case, the infrastructure provider shares its already deployed infrastructure, which ranges from baseband processing units (BBUs) to the RF front ends (also known as Remote Radio Head–RRH). By employing multi-tenancy schemes, operators, MVNOs and verticals can decrease their capital expenditure (CAPEX) and operational expenditure (OPEX). Multi-tenancy makes the deployed infrastructure more energy efficient/greener by allowing a reduced number of BS sites, and, therefore, largely reducing the power consumption of air conditioning and other site support pieces of equipment.

The infrastructure provider can, for example, split its owned spectrum band into smaller chunks and lease it to MNVOs or verticals. Another possible example is instead of splitting its own spectrum into smaller chunks, is the possibility of providing other operators access to their own spectrum bands due to the wide-band capabilities of the current RRHs, ranging from 10 to 250 MHz of useful instantaneous bandwidth [36,37,38]. The virtualization of the physical RRHs is an important step in the direction of multi-tenancy networks as being studied by the 3GPP [39].

**Figure 7:**
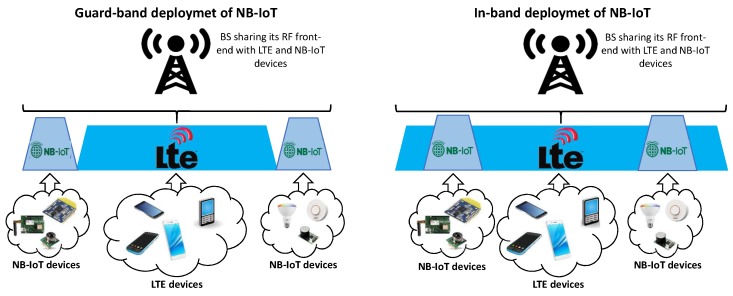
Application of the proposed hypervisor where a single BS serves both LTE and NB-IoT devices/sensors.

This possible application/instantiation of the proposed architecture enables the Radio Access Network (RAN) to be made available as a service (RANaaS) to MVNOs and verticals. Virtual PHYs create new ways for infrastructure providers to monetize their owned spectrum bandwidth and deployed infrastructure. In this way, infrastructure providers can offer virtual PHYs as a service (vPHYaaS) in order to provide isolated and independent virtual networks to MNVOs and/or verticals sitting on the top of a shared physical infrastructure [40]. For this use case, a BBU, providing vPHYs as a service to MNVOs/verticals/operators, would have the proposed hypervisor architecture multiplexing the signal of several vPHY at downlink side while a channelizer would be deployed at uplink side in order to provide each one of the vPHYs with a signal corresponding to its allocated bandwidth, just as depicted in Figure 4. This use case is aligned with standardization efforts made by 3GPP that consider a BS serving both LTE and NB-IoT users (mobile devices and sensors as shown in Figure 7) [40]. This application of the proposed hypervisor allows a single BS to flexibly serve LTE devices and NB-IoT devices (e.g., sensors), decreasing costs and increasing the spectral efficiency by densification.

## 5. Related Work

In this section, we describe and discuss some related pieces of work on virtualization.

The related works on virtualization can be split into two main categories, time- or frequency-multiplexing. The time-multiplexing approaches achieve virtualization by splitting the access time to a common Wi-Fi access-point PHY layer [41,42]. This is possible due to the fact that Wi-Fi is a packet-based wireless network, where access points do not transmit data all the time, and therefore, being able to allow virtual Wi-Fi radio stacks to use its idle time. The frequency-multiplexing approaches can be further divided into two sub-categories based on the type of the virtualized resource, which can be the spectrum bandwidth through the virtualization of the RF front-end [21,22,23,27,43] or the time-frequency resource grid through the virtualization of the resource blocks (RB) provided by Orthogonal Frequency-Division Multiple Access (OFDMA)-based radio technologies such as LTE and WiMax [44,45,46,47].

Out of all the compared related works, only a few are technology agnostic, i.e., can provide virtual radios to any RAT [21,22,23,27,43]. However, the issue with these works is that they exchange optimized performance for being generalized so that they can operate with different RATs. In most of these works [21,22,23,43], in order to be agnostic, the hypervisor layer re-implements operations (e.g., FFT, IFFT, Subcarrier Mapper, etc.) that are already performed by the PHY layers of the individual RATs, creating an extra overhead that decreases the performance of the solution.

An important comparison point is the dynamic creation and destruction of virtual radios without interfering with or stopping the hypervisor or other already instantiated virtual radios. Out of all works, only a few do not support such feature [21,22,23,27,44], where the number of instantiated virtual radios and their respective bandwidth allocations must be configured before running the hypervisor.

Another interesting point of discussion is the maximum possible number of instantiated virtual radios concurrently running during the experiments. The compared works were experimentally tested with the number of concurrently running virtual radios ranging from 1 to 4 virtual radios; however, on the other hand, our prototype has been experimentally tested with 12 concurrently running vPHYs.

Finally, it is also important to add that independent gain configuration is an important feature to be exposed to the virtual radios once, for example, devices might be at totally different locations; however, it seems that independent gain configuration is not a major concern to most of the related works, once only a very few mention its support [23,27,43].

Differently from the other compared works, the virtualization architecture proposed in this work was designed to be highly optimized for sharing the same underlying physical RF front-end among several concurrent multi-carrier-based virtual radios (i.e., vPHYs). It provides dynamic access to several concurrent and configurable virtual RF front ends (e.g., frequency gain, frequency location, and bandwidth) that are accessed through multiple vPHYs. The vPHYs can be instantiated in real time without interfering with running vPHYs and without the necessity to stop the hypervisor.

The majority of the compared related works do not make their source code available [27,41,42,44,45,46,47]; however, we believe that research on this field can only progress if the different implementations are made available for comparison and a better understanding of their functionalities and features. Therefore, we make our proposed architecture prototype available at GitHub [14]. The source code includes some examples to measure the prototype’s performance, a Graphical User Interface (GUI) for visualizing the transmitted spectrum and a channel emulator that can be used to run experiments without the necessity of having a dedicated piece of physical RF front-end. It emulates Additive White Gaussian Noise (AWGN), Rayleigh and Multi-path channels with several different Signal-to-Noise (SNR) values. The prototype makes extensive use of Single Instruction Multiple Data (SIMD) functions, including the FFT and IFFT implementations [48], making it even more optimized.

Table 1 presents a comprehensive comparison of related virtualization and hypervision works.

## 6. Proof of Concept Prototype

In this section, we describe a proof of concept prototype developed to verify the performance of proposed spectrum hypervisor architecture in real-world experiments.

**Figure 8:**
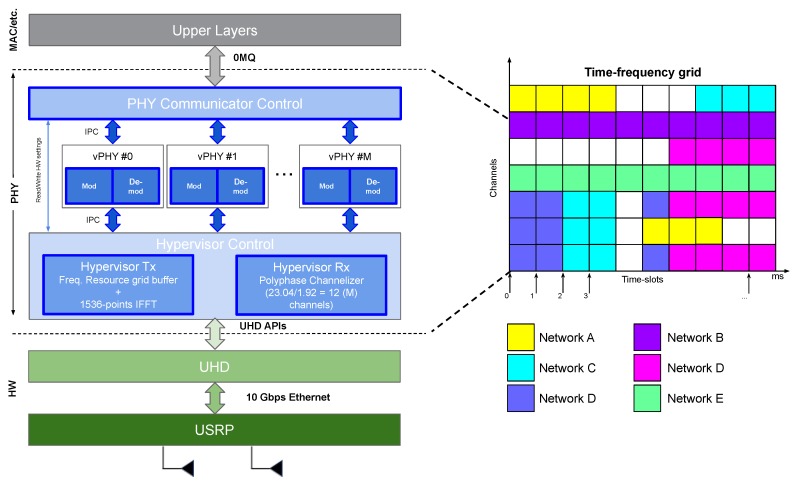
High-level architecture of the implemented prototype.

Figure 8 depicts the high-level architecture of the implementation of the proposed architecture. As can be seen, the prototype is composed of several modules, namely PHY communicator control, *M* vPHYs, and the hypervisor control module, which is in turn, composed of the modules hypervisor Tx and Rx.

The prototype is an open-source software-defined PHY layer designed to multiplex and receive (i.e., demultiplex) the signal of several vPHYs [14]. It is implemented based on the srsLTE library [49]. srsLTE is an open-source and free LTE software-based library [49]. The prototype can run on top of several Ettus software-defined radio (SDR) devices such as the Ettus USRP X family or National Instruments’ (NI) RIO SDR devices [50,51] by using the Universal Software Radio Peripheral (USRP) Hardware Driver (UHD) software Application Programming Interface (API) [17,52]. Therefore, the prototype accesses the SDR device through the UHD driver and its APIs [53].

The communication between the prototype and the upper layers is carried out through a set of three well-defined messages, which are exchanged over a ZeroMQ bus [54], i.e., the prototype and the upper layers are interconnected through a publish-subscribe messaging system known as ZeroMQ [54]. ZeroMQ is a high-performance asynchronous messaging library, designed to be used in distributed or concurrent applications [54]. The set of vPHY messages is designed with Google’s Protocol Buffers (protobuf) [55]. Protobuf is used for data serialization and works perfectly with the 0MQ messaging library [54].

The first two vPHY messages, called, Tx and Rx Control, are used to control and configure the transmission and reception of user data, respectively. The parameters in these two control vPHY messages should be configured and sent to the individual vPHYs by the upper layers before the transmission of every new subframe. Each vPHY control message, as the name suggests, controls the operation of only one vPHY. The remaining message, called Rx statistics vPHY message, is used to give upper layers feedback on the operation of each individual vPHY.

**Tx control** messages transport the user data to be transmitted (i.e., TBs) and transmission parameters such as vPHY ID, vPHY Tx BW, MCS, Tx gain, Tx channel, number of resource blocks used by that user, data length, and data. The vPHY ID field is used in all messages to specify to which one of the vPHYs a control message is meant to or received from. **Rx control** messages are used to configure reception parameters such as vPHY ID, vPHY Rx BW, and Rx channel.

**Rx statistics** messages carry the vPHY ID, received decoded user data, and reception statistics such as Received Signal Strength Indication (RSSI), Channel Quality Indicator (CQI), decoded MCS, cyclic redundancy check (CRC) error counter, etc. The vPHY messages and their parameters are summarized in Table 2.

Next, we describe each one of the modules composing the prototype.

**Figure 9:**
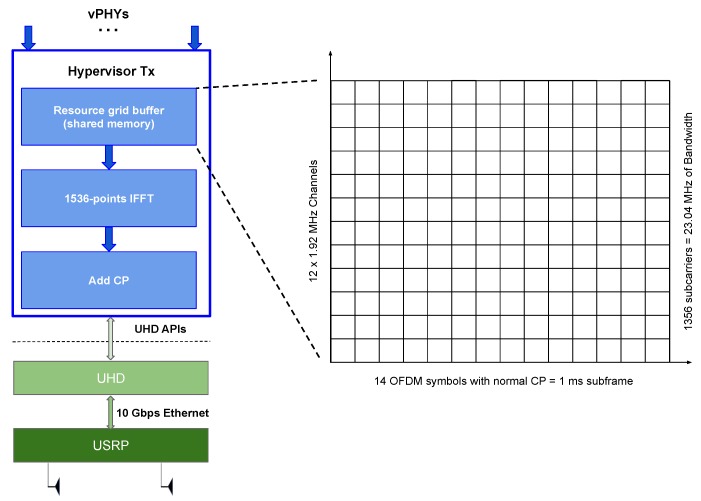
Internal architecture of the Hypervisor Tx module.

- **PHY Communicator Control**: this module is responsible for the exchange of messages with several and possibly independent upper layers, e.g., the MAC layers from different users or operators. This module works on a subframe basis, meaning that the connected upper layers always send/receive in one control message to/from the module the content of a subframe as the minimum unit of data exchange. The received control messages, carrying user data, are then relayed to the respective vPHY by using the vPHY ID in the control message. The decoded user data is sent to the respective upper layer also by using the vPHY ID.
- **vPHY**: modulates and demodulates the user data. After modulating the data, each vPHY maps, according to the channel configured in the Tx control message, its $N_{\mathrm{vPHY}}^{U}$ modulated symbols into a memory buffer, called, *resource grid buffer*, which is a 1 ms (i.e., the duration of a subframe) representation of the frequency-domain spectrum band multiplexed by the PHY prototype. Each vPHY only has to map the $N_{\mathrm{vPHY}}^{U}$ data symbols (i.e., useful symbols), while the remaining positions or subcarriers of the buffer have their values already set to zero before every new transmission. In the current implementation of the prototype, $N_{\mathrm{vPHY}}^{U}=72$ and $N_{\mathrm{vPHY}}=128$ subcarriers. The *resource grid buffer* is a discretized, in number of subcarriers or IFFT points, representation of the spectrum for the duration of 1 ms. In this version of the prototype, we used a 1536-point IFFT. The *resource grid buffer* is a memory buffer that is shared by all vPHYs. In the demodulation case, the IQ samples that are output by the Hypervisor Rx module are decoded accordingly by the respective vPHY. In the current implementation of the prototype 12 vPHYs can be instantiated and concurrently transmit/receive their data. This number of vPHYs is obtained by dividing the number of IFFT points, N=1536, by the total number of vPHY subcarriers including the null ones, $N_{\mathrm{vPHY}}=128$.
- **Hypervisor Tx**: applies a 1536-point IFFT to the *resource grid buffer*, adds CP and transfers the IQ samples to the USRP for transmission over the air. The internal architecture of the Hypervisor Tx module is depicted in Figure 9. As showed in the figure, the Hypervisor Tx can be seen as an OFDM modulator where each OFDM symbol is created by reading and processing the consecutive 1536 data symbols stored at the *resource grid buffer*. As showed in Figure 9, the *resource grid buffer* stores data of 12 channels × 14 OFDM symbols, totaling 1 ms of data.
- **Hypervisor Rx**: applies a FIR polyphase filter-bank channelizer to the IQ samples received from the USRP and outputs the *M* down-converted channels to the vPHYs for data demodulation and decoding. The channels output by the channelizer are centered at 0, $N_{\mathrm{vPHY}}\times \Delta_{f}$, $2\times N_{\mathrm{vPHY}}\times \Delta_{f}$, $3\times N_{\mathrm{vPHY}}\times \Delta_{f}$, *…*, $M-1\times N_{\mathrm{vPHY}}\times \Delta_{f}$, respectively, where $N_{\mathrm{vPHY}}\times \Delta_{f}$ = 1.92 MHz

For improved processing performance, each one of the just described modules runs on an exclusive thread. As the prototype works on a 1 ms basis, it is possible to have a mix of streaming and burst-based transmissions as shown in Figure 8.

The only inconvenience found in the proposed architecture is that it requires that all the involved PHY implementations be jointly modified (although large parts of the implementations suitable for physical RF front ends can be directly reused). This means that in practice, the proposed approach is only valid when the researchers/developers have access to the source code (either open or proprietary source code) of all involved OFDM-based PHYs, since they need to have their code modified in order to be connected to the hypervisor. However, we would like to point out that presently the open-source community offers several full-blown radio stack projects, being the most important and well-known ones srsLTE [49] and Open Air Interface (OAI) [56] projects. Both of them offer mature, well-organized, well-documented, well-written and easily modifiable open-source code for LTE, NB-IoT and 5G-NR radio stacks, including PHY, MAC, RLC, PDCP, RRC, NAS, S1AP and GW layers besides the implementations of EPCs. Additionally, they also have very active mailing lists for researchers/developers to ask questions and report issues with the code.

## 7. Simulation Results

In this section, we present some simulation results carried out to validate and assess the functionality of the proposed architecture.

**Figure 10:**
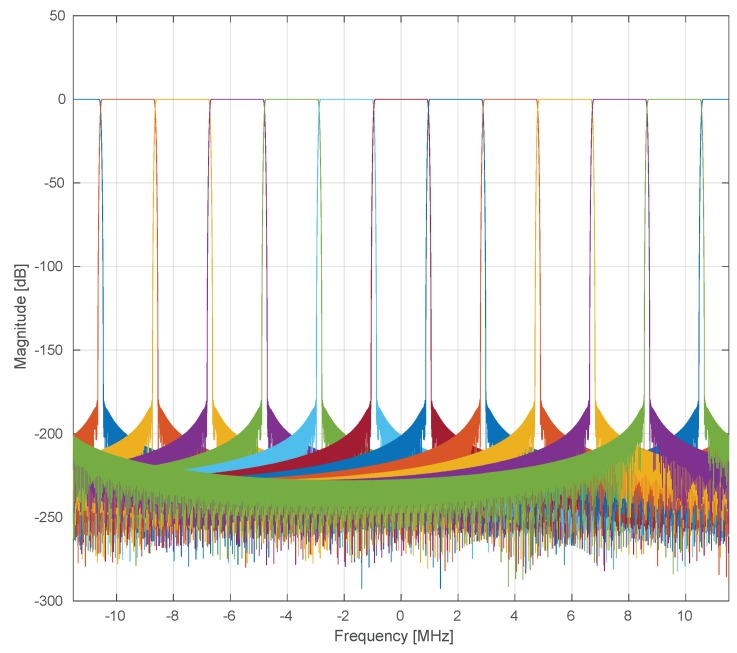
Frequency response of a polyphase filter-bank with stop-band attenuation of 180 [dB] and 512 filter coefficients per sub-band.

For the first validation, we want to check the average Mean Squared Error (MSE) related to the multiplexing of several vPHY modulated signals by the hypervisor. In this simulation, we measure the error between OFDM symbols, $y_{\mathrm{PHY}}$, created with an 128-point IFFT, $N_{\mathrm{PHY}}$, plus 9-sample long CP, $N_{\mathrm{PHY}}^{CP}$, and the OFDM symbols received from the hypervisor after down-conversion and channelization, $y_{\mathrm{vPHY}}$. Each (v)PHY signal modulates 72 consecutive subcarriers, which translates into a useful transmission bandwidth of 1.08 [MHz] when a subcarrier spacing, $\Delta_{f}$, of 15 [KHz] is used. We average the MSE error over $10^{5}$ iterations, where at each iteration, we have the single PHY and all the vPHYs modulated with the same randomly generated data. For this simulation, we use 12 vPHYs, i.e., M=12, where each vPHY has its signals mapped into 128 consecutive subcarriers, $N_{\mathrm{vPHY}}$, totaling 1536 subcarriers, which is the number of points used by the IFFT block in the hypervisor, N. At the receiver side, we use a polyphase FFT analysis filter-bank [18] to split the wide-band signal into multiple uniformly spaced narrow sub-bands. It has a 180 [dB] stop-band attenuation and 512 filter coefficients per sub-band. The frequency response of the polyphase filter-bank used in all the simulations presented in this section is depicted in Figure 10. As can be seen in the figure, the physical spectrum band, $R_{\mathrm{hyper}}$, which is provided by the RF front-end, is split into *M* equal-bandwidth equally spaced sub-bands of 1.92 [MHz] (i.e., $\frac{N\times \Delta_{f}}{M}$). It is important to mention that the stop-band attenuation and filter order parameters play an important role in the fidelity of the multiplexed signals to the single PHY one [18]. The MSE for the *k*-th vPHY is calculated as defined by (13) below.

(13) $$\begin{array}{c} {\mathrm{MSE}}_{k}=E\left[\frac{1}{\left(N_{\mathrm{PHY}}+N_{\mathrm{PHY}}^{CP}\right)}\right.\\ \left.\times \sum_{n=0}^{(N_{\mathrm{PHY}}+N_{\mathrm{PHY}}^{CP})-1}{\left|y_{\mathrm{PHY}}\left(n\right)-y_{\mathrm{vPHY}}\left(n\right)\right|}^{2}\right]. \end{array}$$

We additionally, we have also calculated the average Modulation Error Ratio (MER) to compare the error introduced by the multiplexing to the vPHY modulated signal. The MER compares the error between the modulated data symbols (i.e., the BPSK, QPSK, etc. symbols used to modulate the OFDM subcarriers) and the demodulated data symbols at the receiver side after all the vPHY, hypervisor and channelizer processing. Here we also take the MER average over $10^{5}$ iterations. The MER for the *k*-th vPHY is calculated as follows

(14) $${\mathrm{MER}}_{k}=E\left[10\log_{10}\left(\frac{\sum_{n=0}^{N_{\mathrm{vPHY}}^{U}-1}\left(I_{k}^{2}+Q_{k}^{2}\right)}{\sum_{k=0}^{N_{\mathrm{vPHY}}^{U}-1}{\left(I_{k}-{\tilde{I}}_{k}\right)}^{2}+{\left(Q_{k}-{\tilde{Q}}_{k}\right)}^{2}}\right)\right],$$

where $N_{\mathrm{vPHY}}^{U}$ represents the number of useful subcarriers (i.e., the subcarriers that are modulated with the data symbols), $I_{k}$ is the In-phase value corresponding to the *k*-th reference symbol, $Q_{k}$ is the Quadrature phase value corresponding to the *k*-th reference symbol, ${\tilde{I}}_{k}$ is the In-phase value corresponding to the *k*-th received symbol, and ${\tilde{Q}}_{k}$ is the Quadrature phase value corresponding to the *k*-th received symbol. The MER can be seen as a signal-to-noise ratio (SNR) measurements, where it calculates the distortion/interference caused by the multiplexing operation performed by the hypervisor.

**Figure 11:**
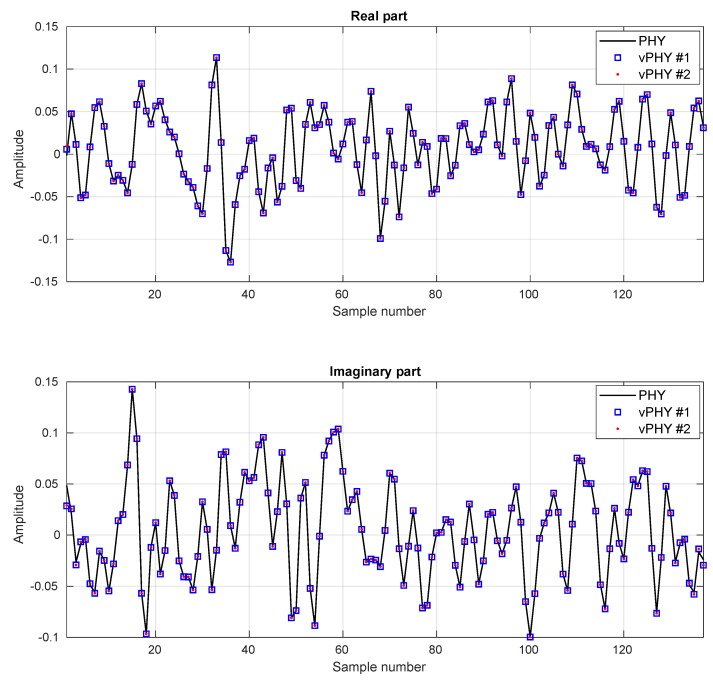
Comparison between OFDM symbols generated by a plain/single PHY and 2 vPHYs.

In Table 3 we show the MSE and MER averages for several different modulation schemes. The MSE estimation values shown in the table were calculated averaging the error between PHY and vPHY OFDM symbols over $1\times 10^{5}$ iterations. The MER averaging was also executed over the same number of iterations. For each iteration, the same set of randomly picked data bits modulates both the single PHY and the 12 vPHYs. The table shows that the MSE is quite low and almost the same for all modulation schemes and that the MER is high and the same for all schemes, which means that both MSE and MER are independent of the employed modulation scheme. Additionally, it is worth mentioning that as all vPHYs are fed with the same set of bits, their resulting MSE and MER are the same, and due to the fact that we only present one value per modulation scheme in Figure 11.

In Table 4 we show the comparison of MSE and MER for several different filter order values. As we noticed with the results in Table 3, the MSE and MER values are almost the same for all modulation schemes, and therefore, in this simulation, we use the same modulation scheme, QPSK, for all trials. As mentioned before, the filter order is important to guarantee a good signal fidelity. This is due to the fact that the higher the filter order, the closer it is to the perfect window filter frequency response, which does not impose any distortion to the filtered signal [57].

In Figure 11, depicts the comparison between OFDM symbols generated by a plain (i.e., single) PHY and 2 vPHYs having their output signals multiplexed by the hypervisor. Here, for the sake of comparison, the single PHY and the 2 vPHYs are fed with the same set of data bits. As can be seen, the vPHY OFDM symbols are quite similar to the OFDM symbol generated by the single PHY.

**Figure 12:**
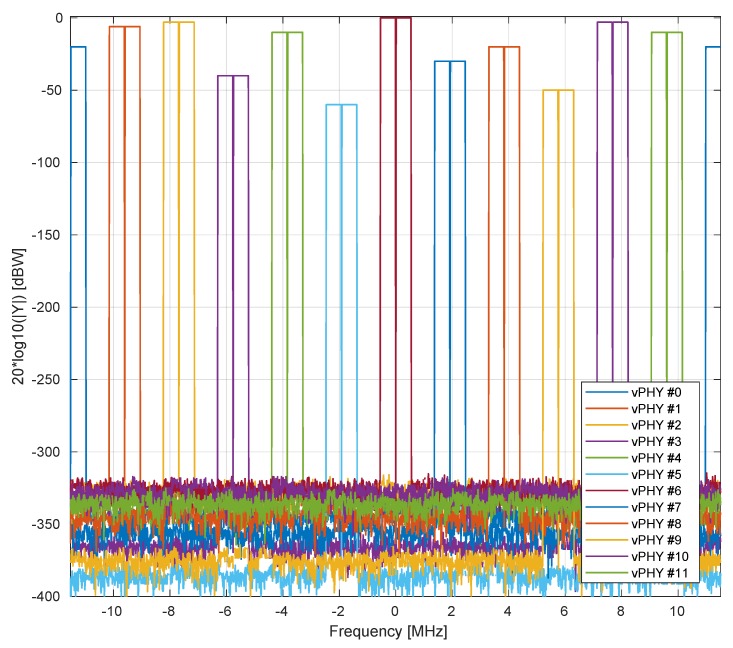
Frequency-domain representation of the wide baseband signal generated by the hypervisor.

**Figure 13:**
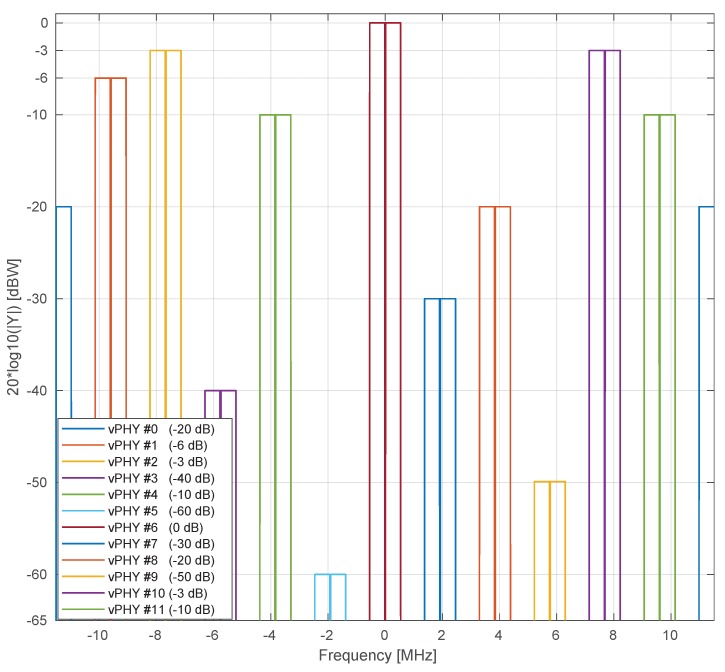
Closer look at the frequency-domain boost factor.

In Figure 12, we show the frequency-domain representation of the wide baseband signal generated by the proposed wireless hypervisor. Here, in this figure, the hypervisor multiplexes, in the frequency domain, the signals of 12 vPHYs, *M*, where each vPHY uses 128 subcarriers, $N_{\mathrm{vPHY}}$, totaling 1536 subcarriers, which is the number of points used by the IFFT block in the hypervisor, N. Each vPHY only modulates 72 subcarriers and leaves the remaining subcarriers along its edges as guard bands. The subcarrier at the center of each vPHY channel is set to 0, which is used to allow receivers to employ simpler/cheap direct-conversion (i.e., zero intermediate-frequency) RF front-end receivers. In the example, shown in the figure, 64QAM modulation is used to modulate the data signal. As can be seen, there are (i) a guard-band between consecutive vPHY signals, (ii) a null-subcarrier exactly at the center of each one of the vPHY transmissions, (iii) and different transmission levels for the vPHYs. As shown in the figure, it is possible to give independent gains to each vPHY, which is accomplished by multiplying the $N_{\mathrm{vPHY}}^{U}$ useful modulation symbols by a multiplication factor varying from 0 (no transmission power at all) to 1 (maximum transmission power used by the physical RF front-end). Figure 13 gives a closer look at the boost factor, showing that it is possible to vary the transmitted power of each individual vPHY just by changing the factor used to multiple the useful subcarriers, $N_{\mathrm{vPHY}}^{U}$. The legend on the figure shows the attenuation given to the default transmission power of a vPHY, which is around 0 [dBW] as shown by the vPHY centered around 0 [Hz].

**Figure 14:**
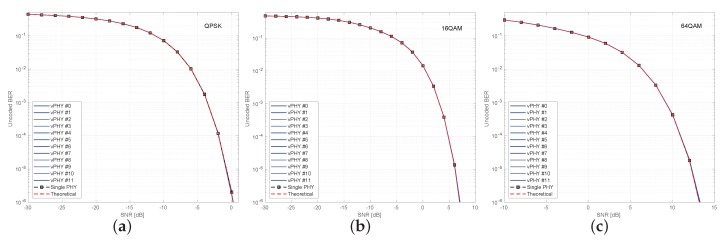
BER curves for a setup where 12 vPHYs concurrently have their signals multiplexed by the hypervisor and use: (**a**) QPSK, (**b**) 16QAM and (**c**) 64QAM modulation schemes.

Figure 14 presents uncoded BER results for a setup where 12 vPHYs concurrently have their signals multiplexed by the hypervisor and employ QPSK, 16QAM, and 64QAM modulation schemes. In each one of the sub-figures, we compare the BER for the 12 vPHYs against the BER achieved by a single PHY, i.e., there is no other signal being transmitted along with that of the PHY under test. In this simulation, we consider an Additive White Gaussian Noise (AWGN) channel. The single PHY and each one of the 12 vPHYs modulate 72 (i.e., $N_{\mathrm{vPHY}}^{U}$) out of 128 (i.e., $N_{\mathrm{vPHY}}$) consecutive subcarriers by using QPSK, 16QAM, and 64QAM modulation schemes. The hypervisor’s IFFT length, *N*, is set to 1536 and the cyclic prefix length, $N_{CP}$, is set to 108 samples. For each SNR point, $1\times 10^{6}$ iterations were run, where the total number of wrongly decoded bits and transmitted bits were calculated for the BER calculation. As can be seen, the vPHY BER curves exactly match the single PHY BER curve (dashed black curve with squares along it), meaning that there is no interference between the current transmissions. Additionally, it is also worth mentioning that all curves match the theoretical BER curves (red-dashed curve with dots along it), which can be approximated by (15) [58].

(15) $$P_{b}\approx \frac{2(\sqrt{\gamma }-1)}{\sqrt{\gamma }\log_{2}\left(\gamma \right)}\mathrm{erfc}\left(\sqrt{\frac{3\log_{2}\left(\gamma \right)\left(E_{b}/N_{0}\right)}{2(\gamma -1)}}\right),$$

where γ is the modulation order and $E_{b}/N_{0}$ is the bit energy over the power spectrum density. The results presented in Figure 14, are very important as they prove that the proposed architecture provides perfect isolation among all the signals being multiplexed. The perfect isolation is due to the orthogonality provided by the IFFT processing in the hypervisor, which guarantees that every single subcarrier, spaced of $\Delta_{f}$ [Hz], is mutually orthogonal to all other ones.

**Figure 15:**
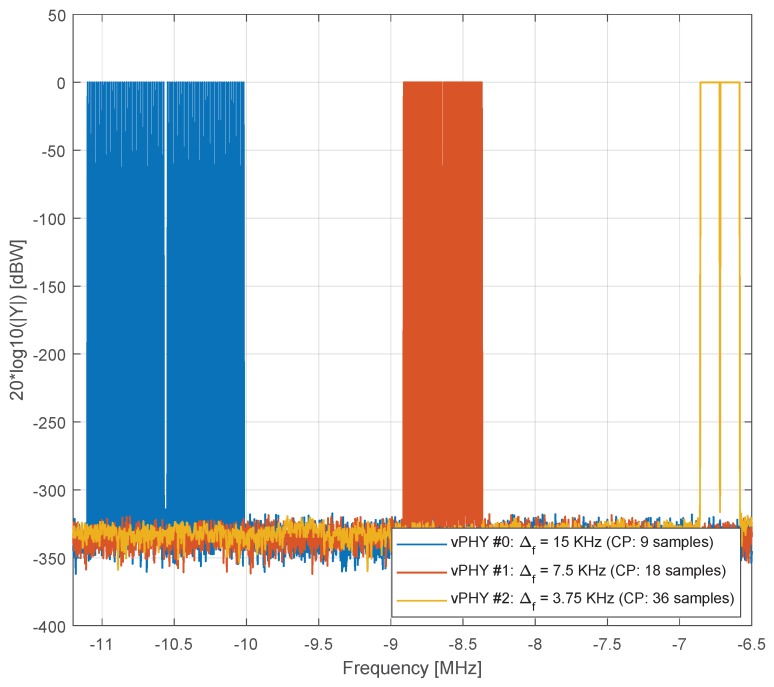
Frequency-domain representation of the wide baseband signal generated by the hypervisor for 3 vPHYs with uncorrelated numerologies.

Figure 15 shows the frequency-domain representation of the wide baseband signal generated by the hypervisor for 3 vPHYs with different but correlated numerologies. These 3 vPHYs employ frequency spacings, $\Delta_{f}$ of 15, 7.5, and 3.75 [KHz], and CP lengths of 9, 18, and 36 samples, FFT/IFFT lengths of 128, 512, and 2048 points, and sampling rates of 1.92, 3.84, and 7.68 [MSps], respectively. In this simulation, we use a hypervisor with an IFFT of 6144 points with a sampling rate of 23.04 [MSps], which results in a subcarrier frequency spacing of 3.75 [KHz]. For all 3 vPHYs, we modulate 72 subcarriers and leave the others unused. As can be seen, even though the 3 vPHYs have only 72 useful subcarriers, we notice that due to the different frequency spacings, $\Delta_{f}$, each one of them has a different occupied bandwidth. In the case of vPHY#0, the useful subcarriers appear every 4 points so that the final subcarrier frequency spacing equals 15 [KHz]. In the case of vPHY#1, the useful subcarriers are mapped into the Hypervisor’s subcarriers every 2 points, resulting in a subcarrier frequency spacing of 7.5 [KHz]. In the case of vPHY#2, the useful subcarriers are mapped consecutively into the Hypervisor’s subcarriers, which results in a subcarrier frequency spacing of 3.75 [KHz]. The receiver side for receiving the signal of vPHY#0 works as before, since we respect the subcarrier frequency spacing of 15 [KHz]. For receiving the signal of the other 2 vPHYs the receiver would need to use FFT with lengths of 512 and 2048 and assume subcarrier frequency spacing of 7.5 and 3.75 [KHz], respectively.

**Figure 16:**
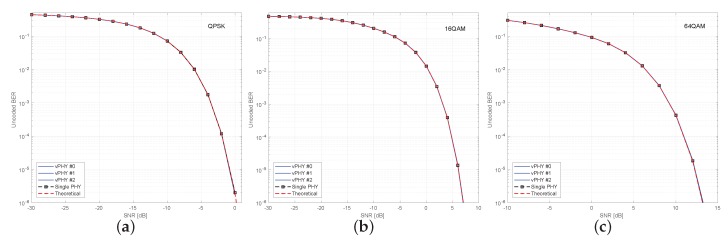
BER curves for a setup with 3 vPHYs with uncorrelated numerologies. Modulations used are (**a**) QPSK, (**b**) 16QAM and (**c**) 64QAM modulation schemes.

Figure 16 shows uncoded BER results for the setup used for plotting Figure 15 where the 3 vPHYs with different but correlated numerologies concurrently have their signals multiplexed by the hypervisor. These 3 vPHYs employ QPSK, 16QAM, and 64QAM modulation schemes for each one of the simulation results presented in the figure. In each one of the sub-figures, we compare the achieved BER of the 3 vPHYs against the BER achieved by a single PHY in AWGN channel. The single PHY and each one of the 3 vPHYs modulate 72 subcarriers by using QPSK, 16QAM, and 64QAM modulation schemes. For each SNR point, $1\times 10^{6}$ iterations were run, where the total number of wrongly decoded bits and transmitted bits were calculated for the BER calculation. As expected, the vPHY BER curves exactly match the single PHY BER curve, meaning that there is no interference between the current transmissions. Additionally, it is important to highlight that even though the vPHYs have different numerologies the Hypervisor is able to multiplex the signals so that the different receivers can successfully decode the signals. Moreover, it is also worth mentioning that all curves match the theoretical BER curves, which are approximated by (15). Again, as expected, the results presented in Figure 16, show that the proposed architecture provides perfect isolation among all the signals being multiplexed, even if they have different but correlated numerologies. This perfect isolation is due to the orthogonality provided by the IFFT processing in the hypervisor, which guarantees that every single subcarrier, spaced of $\Delta_{f}=3.75$ [KHz], is mutually orthogonal to all other ones.

## 8. Experimental Evaluation

In this section, we present some experimental results carried out to validate and assess the functionality of the proof of concept implementation of the proposed architecture. All the experiments presented in this section were carried out with the prototype running on a desktop with an Intel Xeon E5-2650 v4 CPU (@2.2 GHz, 30 M cache, 9.60 GT/s QPI, Turbo, HT, 12 Cores/24 Threads, 105 Watts) with 128 GB of RAM memory connected to a x310 USRP with 10 Gigabit Ethernet link, and equipped with CBX-120 RF daughterboards [59]. These RF daughterboards operate from 1200 up to 6000 MHz with a bandwidth of 120 MHz [59].

For all the experiments presented in this section, each vPHY has a useful transmitting BW of 1.08 MHz, which is equivalent to 6 LTE RBs, and a guard-band of 420 KHz at each side of the transmitted spectrum, totaling 1.92 MHz of used BW (i.e., useful-band plus guard-band sections) per vPHY, totaling 12×1.92 MHz/vPHY =23.04 MHz of occupied BW when we have 12 vPHYs operating concurrently. Due to the lack of full-blown and tested (i.e., operational) open-source projects that implement systems with different but correlated numerologies such as subcarrier frequency spacing, we will not be able to experiment with correlated numerologies. However, as shown in Section 7, working with different but correlated numerologies would not be a problem to the proposed architecture.

**Figure 17:**
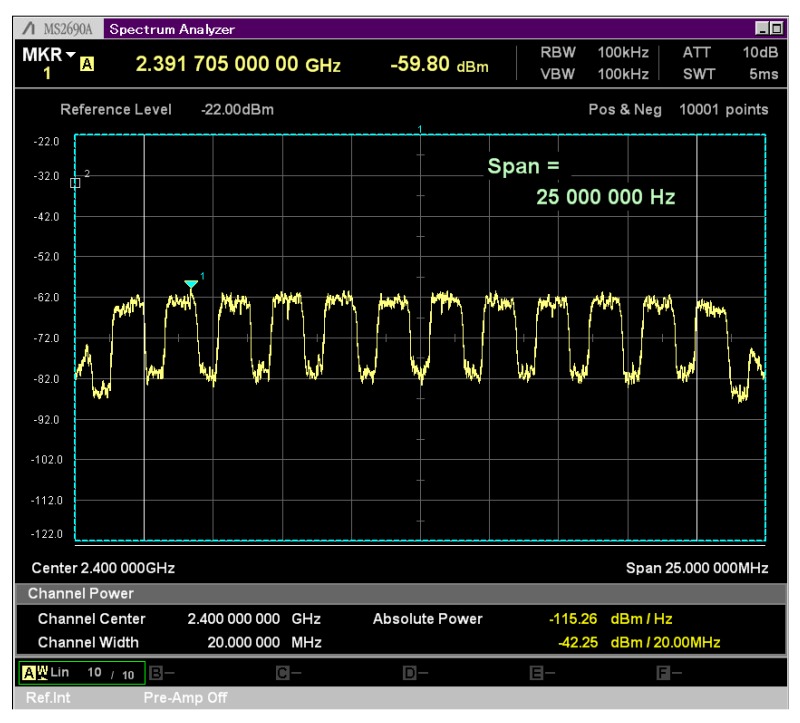
Spectrum of 12 vPHYs concurrently transmitting at a center frequency of 2.4 GHz.

Figure 17 shows the spectrum of 12 vPHYs transmitting concurrently. This figure was collected with an Anritsu MS2690A Signal Analyzer. The RF front-end center frequency was set to 2.4 GHz and Tx gain set to 3 [dB] with the USRP Tx output connected to the signal analyzer through a cable with 20 [dB] of attenuation. As can be seen, the total transmission BW spans over 23.04 MHz, which is equivalent to having 12 1.92 MHz-wide vPHYs with their center frequencies located at 1.92 MHz apart from each other. Additionally, we can also see that the two vPHYs transmitting at the right and left edges suffer from attenuation, which is caused by cascaded integrator-comb (CIC) filter roll-off. CIC filter are implemented in the USRP to provide decimation by an arbitrary programmable integer decimation factor; however, they present a very significant pass-band roll-off, which are often called *spectral droop* or *CIC roll-off* [60].

**Figure 18:**
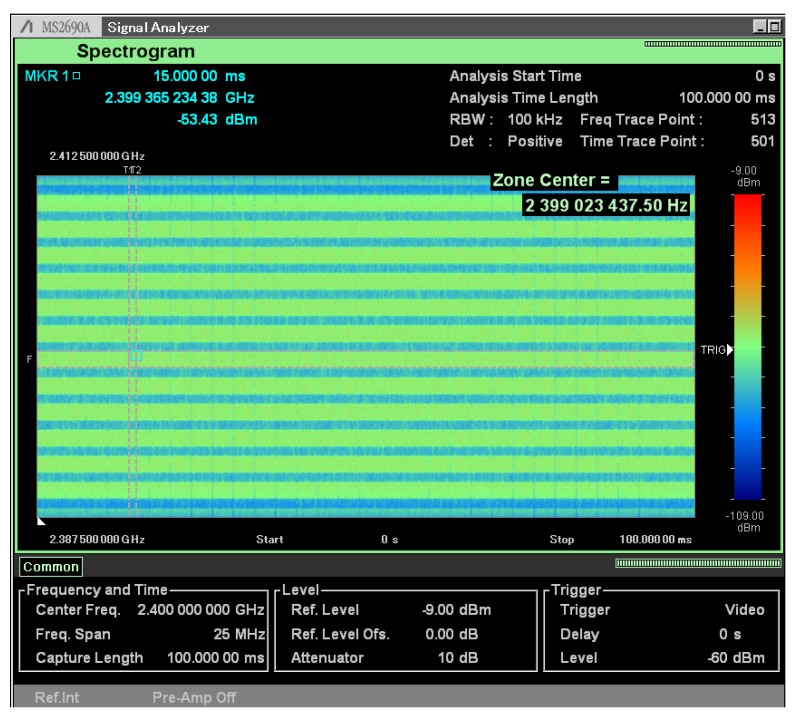
Spectrogram of 12 vPHYs concurrently transmitting at a center frequency of 2.4 GHz.

Figure 18 shows the spectrogram (time versus frequency) for the same experiment setup used to capture Figure 17. In this experiment all 12 vPHYs transmit in streaming mode, i.e., each vPHY transmits subframes all the time with no gap between subsequent subframes. The transmitted signal was captured for a period of 100 ms. It can be noticed that all 12 vPHYs transmit at the same time throughout the whole analysis interval, without any gap between consecutive subframes.

**Figure 19:**
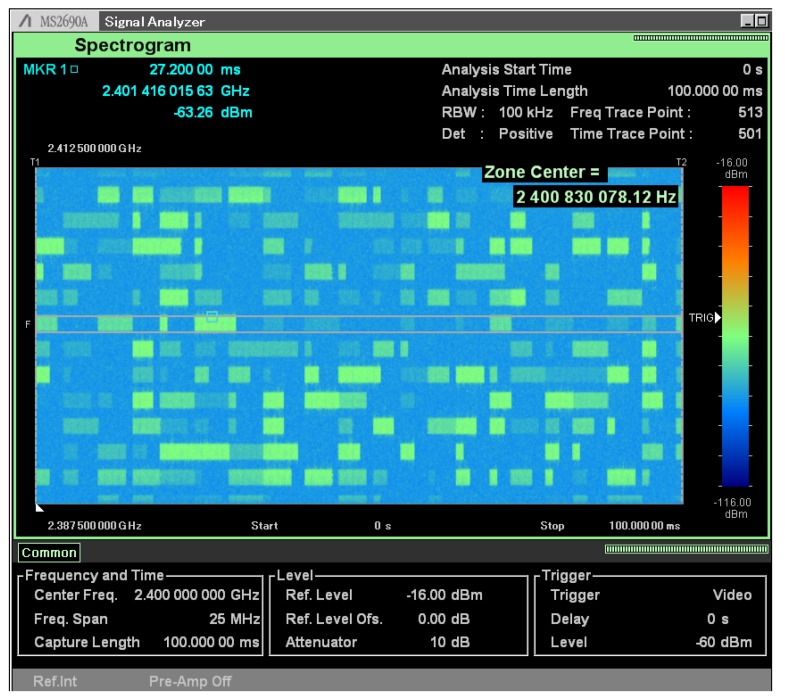
Spectrogram of 12 vPHYs with discontinuous transmissions and independent frequency gains at a center frequency of 2.4 GHz.

Figure 19 shows that the proof of concept prototype of the proposed hypervisor is able to handle discontinuous transmissions and to apply independent gains to each vPHY, as described in Section 3. Again, we used the same experiment setup used to capture Figure 17. In this experiment all 12 vPHYs transmit in discontinuous (burst) mode with random number of subframes transmitted in a row, channel number and frequency amplification factor. The number of subframes, channel number and frequency amplification factor of each vPHY are randomly selected between the ranges 0–11, 0–5, and 0–100% respectively. The transmitted signal was captured for a period of 100 ms. As can be seen, the prototype is also able to work on burst mode with independent frequency amplification factor for each vPHY. This result also shows that the prototype also supports runtime configuration of the number of transmitting vPHYs as we see that not all vPHYs might be transmitting during a period.

The following experiments are executed by adding a channel emulator between the Tx and Rx sides of the prototype. At the Tx side, the generated multiplexed signal, instead of being sent to the USRP HW is sent to an abstraction layer that emulates the HW and adds additive white Gaussian noise (AWGN) to the transmitted signal, next, the abstraction layer transfers the noisy signal to the receiving side of the prototype.

**Figure 20:**
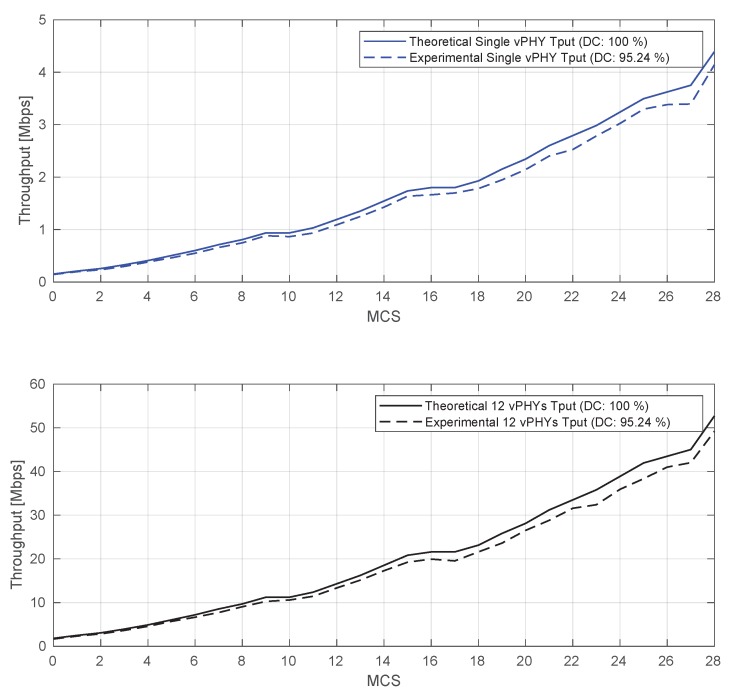
Prototype’s throughput for single and 12 vPHYs over different MCS values.

Figure 20 shows the throughput measurements taken with the proposed architecture prototype for several MCS values and a Duty Cycle (DC) of 95.24%. In this experiment, the prototype works in full-duplex mode (i.e., it is simultaneously transmitting and receiving) with 1 single vPHY (upper part of the figure) and 12 vPHYs (lower part of the figure) working at the same time. We adopt a full-duplex mode to check if this mode somehow impacts the measured throughout, as in full-duplex mode the prototype is being fully used. The measurements were taken with transmissions of 20 ms (i.e., 20 subframes) and a gap of 1 ms between subsequent transmissions, and therefore, a DC of 95.24%. The throughput is calculated as an average over 10 measurement intervals of 10 s each. During one measurement interval (i.e., 10 s) the total number of received bits from all vPHYs is counted and then divided by the interval to produce the throughput measured during that interval. As in this experiment we are only interested in the maximum throughput that can be achieved, the SNR on the link was set to 30 [dB] so that the packet reception rate for all MCS values was equal to 1. For the sake of comparison, the theoretical maximum throughput achieved by the *Streaming* mode (i.e., transmissions with a DC equal to 100%) is added to the figure. The theoretical maximum throughput is calculated by dividing the size in bits of an LTE transport block for each MCS value [61] by 1 ms. As can be seen, the measured prototype’s throughput approaches the theoretical maximum throughput for all MCS values, yielding more than 4.14 Mbps in the single vPHY case and more than 49.2 Mbps in the 12 vPHYs case for MCS 28. Additionally, as can be also noticed, the operation in full-duplex mode has no visible impact on the achieved throughput. This is due to the powerful server, with 12 CPU cores, used to run the prototype.

**Figure 21:**
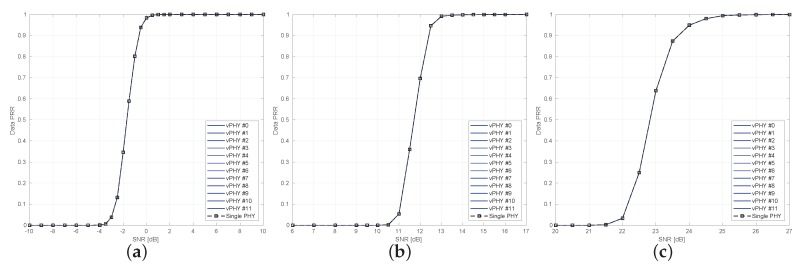
Data PRR curves for a setup with 12 vPHYs with their signals multiplexed by the prototype and using: (**a**) MCS0 (QPSK), (**b**) MCS16 (16QAM), and (**c**) MCS28 (64QAM).

Next, in Figure 21 we compare the data packet reception ratio (PRR) of a single PHY against the data PRR of 12 vPHYs concurrently transmitting over a range of SNR values. In this experiment, 12 vPHYs concurrently have their signals multiplexed, transmitted and received by the prototype. The experiment uses 3 different MCS values so that all 3 modulation schemes employed by LTE standard (i.e., QPSK, 16QAM, and 64QAM) are tested. The PRR is calculated as the average over $10^{5}$ Monte Carlo trials, where at each trial, the Tx side of the prototype sends either a single PHY signal or the multiplexed signal of 12 vPHYs. In each one of the sub-figures, we compare the PRR of the 12 vPHYs against the PRR obtained with a single PHY, i.e., there is no other signal being transmitted along with that of the PHY under test. As theorized earlier, the PRR curve of the 12 concurrent transmitting vPHYs match the PRR of the single PHY (i.e., the dashed black curve with squares along it). This means that there is no interference between the current vPHY transmissions. These results prove that the prototype of the proposed architecture also, as shown before with the simulation results, provides isolation among all the signals being multiplexed by the prototype. The achieved isolation is due to the orthogonality provided by the IFFT processing implemented by the prototype.

**Figure 22:**
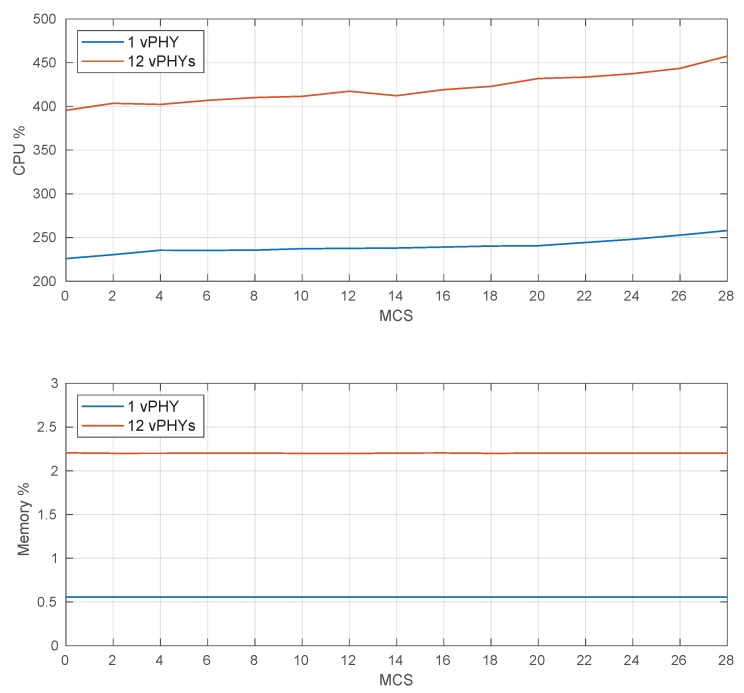
CPU and memory use of the architecture prototype for 1 and 12 vPHYs.

Figure 22 depicts the CPU and memory use of the prototype for several MCS values. These results compare CPU and memory use when the prototype must multiplex the signal of 1 and 12 vPHYs, respectively. The results in the figure were calculated by averaging CPU and memory usage values sampled every 200 ms during the duration of the experiment, which was set to 60 s. Each one of the vPHYs transmit 20 subframes in a row with a 1 ms gap between consecutive transmissions.

As can be observed, the CPU use increases as the MCS increases; however, there is no CPU starvation issue. The increase in CPU use is mainly due to the fact that as the MCS value increases (i.e., higher data rates), the turbo encoding (at Tx side), synchronization and turbo decoding (both at Rx side) processing tasks become more complex and consequently demand a lot more of CPU for data processing. For a MCS equal to 28 and 12 vPHYs concurrently transmitting, the CPU use is of approximately 450%, meaning that the processing power of fewer than 5 cores is being employed, leaving the other cores in the idle state for large periods. On the other hand, we see that the memory use is practically constant for all MCS values and goes from around 0.5% to 2.2% for 1 and 12 vPHYs, respectively. Therefore, memory use is practically independent of the MCS value being used. This is an expected result as all memory being used by the prototype is pre-allocated during its initialization. Therefore, based on the results presented in Figure 22, it can be concluded that the prototype does not exhaust CPU or memory resources as the MCS value increases. These are important results, once they show that given the current server configuration, the prototype can be scaled to support even more vPHYs without exhausting CPU or memory resources.

**Figure 23:**
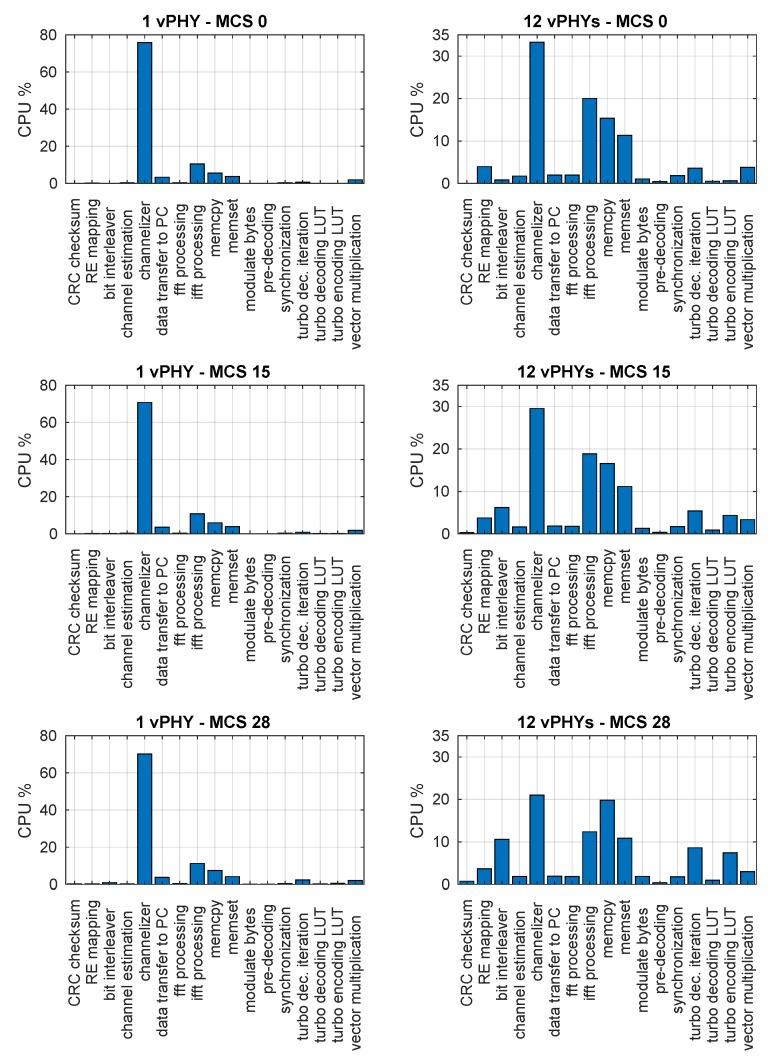
CPU profiling of individual components of the architecture prototype for 1 and 12 vPHYs.

Next, in Figure 23, we present the assessment of the CPU consumption of independent processing tasks making up the architecture prototype. For this assessment we employ the *callgrind* tool, which is part of the *valgrind* profiler. *Callgrind* is a profiling tool that keeps the call history among functions in a program’s run as a call-graph through the use of runtime instrumentation [17,62]. The figure presents the functions with the highest CPU processing load (i.e., the most representative CPU consumers) for 3 different MCS values and the cases where 1 and 12 vPHYs are instantiated. The setup used for this experiment is the same as the one used during the experiment for CPU and memory profiling.

As can be noticed, channelization presents the highest CPU consumption throughout all test cases. Channelization is a quite heavy processing once it keeps always processing a bandwidth equivalent to the maximum number of configured vPHYs, which in this case is equal to 12, no matter the number of actually instantiated vPHYs. For 1 instantiated vPHY, inverse FFT is the second most consuming task; however, its CPU consumption remains constant for all considered MCS values as it does not depend on the MCS used. In the case that 12 vPHYs are instantiated, we see that memory copy, *memcpy*, increases its load from 15.35% for MCS 0 to approximately 20% for MCS 28. Compared to the 1 vPHY case, it is a drastic increase once it has a maximum CPU load of 7.41% for MCS 28. For the 12 vPHYs case, the inverse FFT is the third most consuming task, ranging from 20.04% for a MCS equal to 0 to 12.37% for a MCS equal to 28. We see that in the second column, the one for 12 instantiated vPHYs, the IFFT processing load gradually decreases while *memcpy* gradually increases its CPU load. On the other hand, we also see that the CPU processing load of the memory set operation, *memset*, remains constant throughout the evaluated MCS values. It is also important to highlight that bit interleaving processing gets heavier as the MCS value increases, consuming approximately 0.81% of CPU time for a MCS equal to 0 and going to 10.59% when the MCS value is made equal to 28.

## 9. Conclusions

In this paper, we proposed a wireless spectrum hypervisor architecture that can abstracts a radio frequency (RF) front-end into a configurable number of virtual RF front ends. Our approach was proposed to improve spectral efficiency by efficiently using vacant gaps in congested spectrum bandwidths or employing network densification through infrastructure sharing. We provided a mathematical demonstration on how the proposed approach works and presented several simulation results proving its functionality and efficiency. Additionally, we presented an open-source and free proof of concept prototype of the proposed architecture and several experimental results validating its functionality and showing its performance.

As future work, we highlight the following improvements to the proposed architecture prototype. As we could see, channelization and IFFT (i.e., OFDM modulation) processing tasks consume a lot of CPU time when 12 vPHYs are instantiated, therefore, offloading these tasks to the FPGA can increase both real-time and processing performance. Additionally, memory copy also consumes a lot of CPU time, and therefore, smarter ways of carrying out these copies should be investigated. The proposed architecture supports only multi-carrier-based waveforms; however, it would be interesting to add support to other kinds of waveforms, and therefore, another direction would be adding the support to non-multi-carrier waveforms. Finally, the integration of the proposed architecture’s prototype with LTE or 5G-like upper layers is a direct sequence of the work presented here and would serve as a demonstration of what can be achieved with the proposed architecture in terms of real deployments.

## Figures and Tables

**Table 1:** Comparison of related works.

	Related Work										
**Comparison Metric**	This work	[23]	[43]	[44]	[41]	[45]	[27]	[46]	[47]	[21]	[42]
Multiplexing domain	Frequency	Frequency	Frequency	Frequency	Time	Frequency	Frequency	Frequency	Frequency	Frequency	Time
Virtual resource type	Spectrum	Spectrum	Spectrum	RB	AP in time	RB	Spectrum	RB	RB	Spectrum	AP in time
RAT agnostic	Only MC ${}^{10}$	Yes	Yes	Only WiMax	Only Wi-Fi	Only LTE	Yes	Only WiMax/LTE	Only LTE	Yes	Only Wi-Fi
Spectrum flexibility ${}^{11}$	Yes	Yes	Yes	Yes	FC/FB ${}^{12}$	Yes	Yes	Yes	Yes	Yes	FC/FB ${}^{12}$
Operation mode	S/PB	S	S	S	PB	S	S	S	S	S	PB
Simulation validated	Yes	No	No	Yes	No	No	No	No	Yes	No	No
Experimentally validated	Yes	Yes	Yes	Yes	Yes	Yes	Yes	No	No	Yes	Yes
Implementation	SW	SW	SW	SW	SW	SW	SW	SW	SW	SW	SW
Create self-interference	No ${}^{1}$	Yes	Yes	No ${}^{1}$	No ${}^{2}$	No	Yes	No	No	Yes	No ${}^{2}$
Create OOB emissions	Yes	Yes	Yes	No	Yes	No	Yes	No	No	Yes	Yes
Individual gain control	Yes	Yes	Yes	NA	No	No	Yes	NA	NA	No	No
Open-source prototype	Yes	Yes	Yes	No	No	No	No	No	No	Yes	No
Dynamic allocation ${}^{3}$	Yes	No	Yes	NA	Yes	Yes	No	Yes	Yes	No	Yes
Radio resource isolation ${}^{4}$	Yes	Yes	Yes	Yes	Yes	Yes	Yes	Yes	Yes	Yes	Yes
Virtual radio independence ${}^{5}$	Yes	No	Yes	NA	Yes	Yes	Yes	Yes	Yes	Yes	Yes
Concurrent Tx/Rx at different channels	Yes	Yes	Yes	Yes	No	No ${}^{6}$	Yes	No ${}^{6}$	No ${}^{6}$	Yes	No
Number of VR instances ${}^{7}$	12	2	2	2 slices ${}^{9}$	2 ${}^{8}$	3 slices ${}^{9}$	3	NA	4 slices ${}^{9}$	4	2 ${}^{8}$

**Legend**: RB: resource-block–MC: multi-carrier–AP: access-point–SW: software–S: streaming–PB: packet-based–NA: information not-available–VR: virtual radio–FC: fixed channel center frequency–FB: fixed channel bandwidth.${}^{1}$ OFDM-based systems do not create self-interference as the subcarriers are mutually orthogonal. ${}^{2}$ Once it time-multiplexes the access-point. ${}^{3}$ Allow on demand destruction and creation of VRs without interrupting the operation of the spectrum hypervisor or other VRs. ${}^{4}$ Allocate non-overlapping sub-bands to different VRs and prevent interference among VRs, e.g., guard bands. ${}^{5}$ Ensure that VRs cannot interfere with the operation and performance of other VRs, even in the case of a malfunctioning or misbehaving VRs. ${}^{6}$ Use the concept of virtual RBs, therefore, concurrent transmissions only happen at different RBs within the same channel. ${}^{7}$ The maximum number of VRs instantiated during the experiments. ${}^{8}$ A single AP PHY that is time-shared between two VRs. ${}^{9}$ A single PHY layer that has its RBs split into slices, creating VRs. ${}^{10}$ Optimized for multi-carrier-based waveforms, e.g., OFDM. ${}^{11}$ Flexibility in setting different channel center frequencies and bandwidths to different concurrent VRs. ${}^{12}$ The approach only works with fixed channel center frequencies and bandwidths as it time-multiplexes a Wi-Fi AP.

**Table 2:** List of messages and their respective parameters.

vPHY Message	Parameter	Type	Unit	Range
**Tx control**	vPHY ID	uint32	-	0–1
	Tx gain	float	%	0-100%
	Tx vPHY BW	uint8	MHz	1–6 ^1^
	Tx channel	uint32	-	≥0
	Data struct	uchar[]	-	uchar range
	MCS	uint8	-	0–28
	# of resource blocks	uint8	-	1–100
	User Data length	uint32	-	>0
	User data	uchar[]	-	uchar range
**Rx control**	vPHY ID	uint32	-	0–1
	Rx vPHY BW	uint8	MHz	1–6 ^1^
	Rx channel	uint32	-	≥0
**Rx statistics**	vPHY ID	uint32	-	0–1
	CQI	uint8	-	0–15
	RSSI	float	dBW	float range
	Noise	float	dBW	float range
	Decoded MCS	uint8	-	0–28
	CRC error counter	uint32	-	≥0
	Data length	uint32	-	≥0
	Received data	uchar[]	-	uchar range

${}^{1}$ These numbers correspond to the following LTE bandwidths: 1.4, 3, 5, 10, 15 and 20 MHz respectively.

**Table 3:** MSE and MER for several different modulation schemes.

	Modulation Order					
	BPSK	QPSK	16QAM	64QAM	128QAM	256QAM
**MSE**	3.8517 $\times \;10^{-10}$	3.8521 $\times \;10^{-10}$	3.8533 $\times \;10^{-10}$	3.8526 $\times \;10^{-10}$	3.8514 $\times \;10^{-10}$	3.8526 $\times \;10^{-10}$
**MER [dB]**	70.572	70.572	70.572	70.572	70.572	70.572

**Table 4:** MSE and MER for several different filter orders.

Filter Order						
	16	64	128	256	512	1024
**MSE**	1.3317 $\times \;10^{-3}$	1.9853 $\times \;10^{-8}$	3.6452 $\times \;10^{-9}$	3.2091 $\times \;10^{-9}$	3.8513 $\times \;10^{-10}$	2.3038 $\times \;10^{-10}$
**MER [dB]**	5.1897	53.4555	60.8164	61.3699	70.5720	72.8092
